# Regulatory Roles of *Drosophila* Insulin-Like Peptide 1 (DILP1) in Metabolism Differ in Pupal and Adult Stages

**DOI:** 10.3389/fendo.2020.00180

**Published:** 2020-04-21

**Authors:** Sifang Liao, Stephanie Post, Philipp Lehmann, Jan A. Veenstra, Marc Tatar, Dick R. Nässel

**Affiliations:** ^1^Department of Zoology, Stockholm University, Stockholm, Sweden; ^2^Department of Ecology and Evolutionary Biology, Brown University, Providence, RI, United States; ^3^Institut de Neurosciences Cognitives et Intégratives d'Aquitaine (CNRS UMR5287), University of Bordeaux, Pessac, France

**Keywords:** insulin signaling, development, metabolism, respirometry, stress responses, adult tissue growth

## Abstract

The insulin/IGF-signaling pathway is central in control of nutrient-dependent growth during development, and in adult physiology and longevity. Eight insulin-like peptides (DILP1–8) have been identified in *Drosophila*, and several of these are known to regulate growth, metabolism, reproduction, stress responses, and lifespan. However, the functional role of DILP1 is far from understood. Previous work has shown that *dilp1*/DILP1 is transiently expressed mainly during the pupal stage and the first days of adult life. Here, we study the role of *dilp1* in the pupa, as well as in the first week of adult life, and make some comparisons to *dilp6* that displays a similar pupal expression profile, but is expressed in fat body rather than brain neurosecretory cells. We show that mutation of *dilp1* diminishes organismal weight during pupal development, whereas overexpression increases it, similar to *dilp6* manipulations. No growth effects of *dilp1* or *dilp6* manipulations were detected during larval development. We next show that *dilp1* and *dilp6* increase metabolic rate in the late pupa and promote lipids as the primary source of catabolic energy. Effects of *dilp1* manipulations can also be seen in the adult fly. In newly eclosed female flies, survival during starvation is strongly diminished in *dilp1* mutants, but not in *dilp2* and *dilp1*/*dilp2* mutants, whereas in older flies, only the double mutants display reduced starvation resistance. Starvation resistance is not affected in male *dilp1* mutant flies, suggesting a sex dimorphism in *dilp1* function. Overexpression of *dilp1* also decreases survival during starvation in female flies and increases egg laying and decreases egg to pupal viability. In conclusion, *dilp1* and *dilp6* overexpression promotes metabolism and growth of adult tissues during the pupal stage, likely by utilization of stored lipids. Some of the effects of the *dilp1* manipulations may carry over from the pupa to affect physiology in young adults, but our data also suggest that *dilp1* signaling is important in metabolism and stress resistance in the adult stage.

## Introduction

The insulin/IGF signaling (IIS) pathway plays a central role in nutrient-dependent growth control during development, as well as in adult physiology and aging (1–5). More specifically, in mammals, insulin, IGFs, and relaxins act on different types of receptors to regulate metabolism, growth, and reproduction (6–9). This class of peptide hormones has been well conserved over evolution and therefore the genetically tractable fly *Drosophila* is an attractive model system for investigating IIS mechanisms (1, 10, 11). Eight insulin-like peptides (DILP1–8), each encoded on a separate gene, have been identified in *Drosophila* (11–14). The genes encoding these DILPs display differential temporal and tissue-specific expression profiles, suggesting that they have different functions (12, 13, 15–17). Specifically, DILP1, 2, 3, and 5 are mainly expressed in median neurosecretory cells located in the dorsal midline of the brain, designated insulin-producing cells (IPCs) (12, 17–20). The IPC-derived DILPs can be released into the open circulation from axon terminations in the corpora cardiaca, the anterior aorta, the foregut, and the crop. Genetic ablation of the IPCs reduces growth and alters metabolism, and results in increased resistance to several forms of stress and prolongs lifespan (19, 21).

The functions of the individual DILPs produced by the IPCs may vary depending on the stage of the *Drosophila* life cycle. Already, the temporal expression patterns hint that DILP1–3 and 5 play different roles during development. Thus, whereas DILP2 and 5 are relatively highly expressed during larval and adult stages, DILP1 and 6 are almost exclusively expressed during pupal stages under normal conditions (16, 22).

DILP1 is unique among the IPC-produced peptides since it can be detected primarily during the pupal stage (a non-feeding stage) and the first few days of adult life when residual larval/pupal fat body is present (16, 17). Furthermore, in female flies kept in adult reproductive diapause, where feeding is strongly reduced, *dilp1*/DILP1 expression is also high (17). The temporal expression profile of *dilp1*/DILP1 resembles that of *dilp6*/DILP6 although the latter peptide is primarily produced by the fat body, not IPCs (16, 22). Since DILP6 was shown to regulate growth of adult tissues during pupal development (16, 22), we asked whether also DILP1 plays a role in growth control. It is known that overexpression of several of the DILPs is sufficient to increase body growth through an increase in cell size and cell number, and especially DILP2 produces a substantial increase in body weight (12, 23, 24). In contrast, not all single *dilp* mutants display a decreased body mass. The *dilp1, dilp2*, and *dilp6* single mutants display slightly decreased body weight (11, 16, 22), whereas the *dilp3, dilp4, dilp5*, and *dilp7* single mutants display normal body weight (11). However, a triple mutation of *dilp2, 3*, and *5* causes a drastically reduced body weight, and a *dilp1–4,5* mutation results in a further reduction (11, 25). Note that several of the above studies do not show *bona fide* effects on cell or organismal growth (e.g., volume or cell numbers/sizes); they only provide body mass data.

There is a distinction between how DILPs act in growth regulation. DILPs other than DILP1 and DILP6 promote growth primarily during the larval stages (both feeding and wandering stages) when their expression is high (12, 23). This nutrient-dependent growth is relatively well-understood and is critical for production of the steroid hormone ecdysone and thereby developmental timing and induction of developmental transitions such as larval molts and pupariation (26–30). The growth in the pupal stage, which primarily affects imaginal discs and therefore adult tissues, is far less studied [see Slaidina et al. (16) and Okamoto et al. (31)]. In this study, we investigate the role of *dilp1*/DILP1 in growth regulation in *Drosophila* in comparison to *dilp6*/DILP6. For this, we determine both *bona fide* size of body and/or wings and provide wet weights, and thus can distinguish between growth and increase of body mass. We found that mutation of *dilp1* diminishes body weight (but not body size), whereas ectopic *dilp1* expression promotes organismal growth by increasing both weight and size during the pupal stage, similar to *dilp6*. Thus, we cannot unequivocally show a role of *dilp1* in organismal growth, but it does regulate body mass, suggesting that *dilp1* affects metabolism and energy stores. Determination of metabolic rate (MR) and respiratory quotient (RQ) as well as triacylglyceride (TAG) levels during late pupal development provides evidence that *dilp1* and *dilp6* increase the MR and that the associated increased metabolic cost is fueled by increased lipid catabolism.

Since *dilp1*/DILP1 levels are high the first week of adult life, we also investigated the role of *dilp1* mutation and overexpression on early adult physiology, including metabolism stress resistance and fecundity. Interestingly, the newly eclosed *dilp1* mutant flies are less resistant to starvation than controls and *dilp2* mutants. Thus, *dilp1* acts differently from other *dilps* for which it has been shown that reduced signaling increases survival during starvation (21). Also, early egg laying and female fecundity are affected by *dilp1* overexpression, and in general, *dilp1* manipulations produce more prominent effects in female flies.

Taken together, our data suggest that ectopic expression of *dilp1*/DILP1 promotes growth of adult tissues during the pupal stage, and that this process mainly utilizes stored lipids to fuel the increased MR. The DILP1 signaling also affects the metabolism in the young adult fly, and we see sex dimorphic effects of altered signaling on stress responses and fecundity.

## Methods

### Fly Lines and Husbandry

Parental flies were reared and maintained at 18°C with 12:12 light:dark cycle on food based on a recipe from Bloomington *Drosophila* Stock Center (BDSC) (https://bdsc.indiana.edu/information/recipes/bloomfood.html). The experimental flies were reared and maintained at 25°C, with 12:12 light:dark cycle on an agar-based diet with 10% sugar and 5% dry yeast.

The following Gal4 lines were used in this study: *dilp2*-Gal4 [(19) from E. Rulifson, Stanford, CA], *ppl*-Gal4 [(32) from M. J. Pankratz, Bonn, Germany], *To*-Gal4 [(33) from B. Dauwalder, Houston, TX], *c929*-Gal4 [(34) from Paul H. Taghert], yw; UAS-*dilp6*, and yw; UAS-*dilp2;*+ [(23) from H. Stocker, Zürich, Switzerland]. Several UAS-*dilp1* lines were produced for a previous study (35), and two of them, UAS-*dilp1* (II) and UAS-*dilp1* (III), were used here. UAS-*dilp1*-RNAi flies were from Vienna *Drosophila* Resource Center (VDRC), Vienna, Austria. As controls, we used *w*^1118^ or *yw* obtained from BDSC, crossed to Gal4 and UAS lines. All flies (except yw; UAS-*dilp6*, and yw; UAS-*dilp2;* +) were backcrossed to *w*^1118^ for at least 6 generations.

We used a double null mutation of *dilp1/dilp2* that was previously generated by homologous recombination and verified as described by Post et al. (35). Also, single *dilp1* and *dilp2* null mutants were employed. We refer to these three null mutants as *dilp1, dilp2*, and *dilp1/dilp2* mutants for simplicity. As described earlier (35), these were obtained from BDSC and a residual w+ marker was Cre excised followed by chromosomal exchange to remove *yw* markers on chromosomes 2 and X.

To generate a recombinant *dilp6;;dilp1* double mutant, the *dilp1* and *dilp6*^68^ mutants (11) were used for crossing with a double balancer fly, 4E10D/FM7,dfd;;Vno/TM3,dfd, obtained from Dr. Vasilios Tsarouhas (Stockholm University). The efficiency of the *dilp6;;dilp1* double mutant was validated by qPCR.

### Antisera and Immunocytochemistry

For immunolabeling, tissues from larvae or female adults were dissected in chilled 0.1 M phosphate buffered saline (PBS). They were then fixed for 4 h in ice-cold 4% paraformaldehyde (PFA) in PBS, and subsequently rinsed in PBS three times for 1 h. Incubation with primary antiserum was performed for 48 h at 4°C with gentle agitation. After rinse in PBS with 0.25% Triton-X 100 (PBS-Tx) four times, the tissues were incubated with secondary antibody for 48 h at 4°C. After a thorough wash in PBS-Tx, tissues were mounted in 80% glycerol with 0.1 M PBS.

The following primary antisera were used: Rabbit or guinea pig antiserum to part of the C-peptide of DILP1 diluted 1:10,000 (17). Rabbit antisera to A-chains of DILP2 and DILP3 (36) and part of the C-peptide of DILP5 (37) all at a dilution of 1:2,000, mouse anti-green fluorescent protein (GFP) at 1:000 (RRID: AB_221568, Invitrogen, Carlsbad, CA). The following secondary antisera were used: goat anti-rabbit Alexa 546, goat anti-rabbit Alexa 488, and goat anti-mouse Alexa 488 (all from Invitrogen). Cy3-tagged goat anti-guinea pig antiserum (Jackson ImmunoResearch, West Grove, PA). All were used at a dilution of 1:1,000.

### Image Analysis

Images were captured with a Zeiss LSM 780 confocal microscope (Jena, Germany) using 10 ×, 20 ×, and 40 × oil immersion objectives. The projections of z-stacks were processed using Fiji (https://imagej.nih.gov/ij/). The cell body outlines were extracted manually and the staining intensity was determined using ImageJ (https://imagej.nih.gov/ij/). The background intensity for all samples was recorded by randomly selecting three small regions near the cell body of interest. The final intensity value of the cell bodies was determined by subtracting the background intensity.

Images of pupae, adult flies, and fly wings were captured with a Leica EZ4HD light microscope (Wetzlar, Germany). The size of the adult fly body and wings was determined using Fiji. The pupal volume (*v*) was calculated using the equation *v* = 4/3 π (*L*/2) × (*l*/2)^2^, in which *L* = length and *l* = width (38). Thorax length was measured from the posterior tip of the scutellum to the base of the most anterior point of the humeral bristle.

### Pupariation Time, Egg to Pupae Viability, and Adult Body Weight

To determine time to pupariation, 6- to 7-day-old adult females were crossed in the evening. The following morning, adult flies were transferred to vials with fresh food on which they were allowed to lay eggs for 4 h. Two hours after the initiation of egg laying was considered time “0,” and thereafter, the number of pupae was monitored at 6- or 12-h intervals. To investigate the viability of egg to pupae formation, one pair of 6- to 7-day-old adult flies was allowed to lay eggs for 24 h, after which the total number of eggs was counted. Subsequently, the total number of pupae was counted and the viability of egg to pupae was determined as pupa number/egg number × 100%. The body weight (wet weight) of single adult flies was determined using a Mettler Toledo MT5 microbalance (Columbus, USA). The number of eggs of stage 10–14 in ovaries was counted in 3-day-old flies.

### Starvation and Desiccation Survival Assay

Newly eclosed and mated 6- to 7-day-old adults were used for starvation and desiccation resistance experiments. For newly eclosed flies, we collected virgin flies every 4 h, to be used for starvation experiments. The flies were kept in vials containing 5 ml of 0.5% aqueous agarose (A2929, Sigma-Aldrich) for starvation and empty vials for desiccation. The number of dead flies was counted at least every 12 h until all the flies were dead. At least 110 flies from three replicates were used for the analysis.

### Capillary Feeding (CAFE) Assay

Food intake was measured using a slightly modified CAFE assay following Ja et al. (39). In brief, female flies were placed into 1.5-ml Eppendorf microcentrifuge tubes with an inserted capillary tube (5 μl, Sigma-Aldrich) containing 5% sucrose, 2% yeast extract, and 0.1% propionic acid. To estimate evaporation, three food-filled capillaries were inserted in identical tubes without flies. The final food intake was determined by calculating the decrease in food level minus the average decrease in the three control capillaries. Food consumption was measured daily and calculated cumulatively over 4 consecutive days. For this assay, we used 8–10 flies in each of three biological replicates.

### Quantitative Real-Time PCR (qPCR)

Total RNA was extracted from whole bodies of middle or late pupal stages of *Drosophila* by using Trizol-chloroform (Sigma-Aldrich). Quality and concentration of the RNA were determined with a NanoDrop 2000 spectrophotometer (Thermo Scientific). The concentration of the RNA was adjusted to 400 ng/μl. A total of 2 μg RNA was used for cDNA synthesis. The cDNA syntheses were performed by using random hexamer primer (Thermo Scientific) and RevertAid reverse transcriptase (Thermo Scientific). The cDNA products were then diluted 10 times and applied for qPCR using a StepOnePlus^TM^ instrument (Applied Biosystems, USA) and SensiFAST SYBR Hi-ROX Kit (Bioline) following the protocol from the manufacturer. The mRNA abundance was normalized to ribosomal protein (rp49) levels in the same samples. Relative expression values were determined by the 2^−ΔΔCT^ method (40). The sequences of primers used for qPCR were those used previously (17, 35, 41):

dilp1 F: CGGAAACCACAAACTCTGCG
dilp1 R:CCCAGCAAGCTTTCACGTTT
dilp2 F: AGCAAGCCTTTGTCCTTCATCTC
dilp2 R: ACACCATACTCAGCACCTCGTTG
dilp3 F: TGTGTGTATGGCTTCAACGCAATG
dilp3 R: CACTCAACAGTCTTTCCAGCAGGG
dilp6 F: CCCTTGGCGATGTATTTCCCAACA
dilp6 R: CCGACTTGCAGCACAAATCGGTTA
rp49 F: ATCGGTTACGGATCGAACAA
rp49 R: GACAATCTCCTTGCGCTTCT.

### Metabolite Quantification

Glycogen and TAG levels were assayed as previously described (35, 42, 43). For glycogen assays, 5–6 adult female flies per sample were homogenized in PBS and quantified using the Infinity Glucose Hexokinase reagent by spectrophotometry. For TAG assays, 5–6 adult female flies per sample were homogenized in PBS + 0.05% TBS-T and quantified using the Infinity Triglycerides reagent by spectrophotometry. The fly lysate protein levels were determined by BCA assay (Thermo Fisher) and metabolite levels were normalized to protein level.

To measure the amount of TAG during late pupal stages, 6 replicates with 4 pupae in each were collected and then homogenized in PBS + 0.05% Triton X-100 with a tissuelyser II from Qiagen. The TAG levels were determined with a Liquick Cor-TG diagnostic kit (Cormay, Poland) using a linear regression coefficient from a standard curve made with 2.2 μg/μl TAG standard (Cormay, Poland). Absorbance of samples was measured at 550 nm with a micro-plate reader (Thermo scientific). Data are expressed as micrograms of TAG related to protein levels. Protein levels were determined using a Bradford assay according to Diop et al. (44).

### Dynamic Injection Respirometry

Carbon dioxide (CO_2_) production and oxygen (O_2_) consumption of individual pupae of both sexes were measured during pupal development at 25°C to assess MR as described previously (45). Pupae were placed in 1-ml syringes (i.e., respirometry chambers) that were filled with air scrubbed of CO_2_ with ascarite (Acros Organics, USA) that then passed through filtered acidified water (pH <4.5, checked weekly), closed with three-way luer valves, and kept for roughly 24 h at 25°C with 12:12 light:dark cycle. An empty syringe served as control. CO_2_ production was measured using a Sable Systems (Las Vegas, NV, USA) differential respirometry setup. Two independent lines of outdoor air scrubbed of H_2_O and CO_2_, using drierite (WA Hammond Drierite, USA) and ascarite scrubbers, respectively, were pushed at a steady rate of 150 ml min^−1^ using as SS-4 pump (Sable Systems) and two separate mass flow controllers (840 Series; Sierra Instruments Inc., California, USA). The syringes containing pupae were placed after the mass valve controllers in the first line (sample) and 0.45 ml pushed into the airflow. The push rate was recorded through a second flow meter downstream of the syringe and approximated a flow rate of 162 ml min^−1^ downstream of the syringe. The line was then scrubbed of H_2_O with magnesium perchlorate (Sigma-Aldrich) and entered the sample line of a Li-7000 CO_2_ analyzer (LiCor, Lincoln, NE, USA). The second line (reference) proceeded the same way, mimicking the exact length of the sample line (including an empty measurement chamber), entering the reference line of the CO_2_ analyzer. The lines then proceeded through a second set of ascarite CO_2_ scrubbers and entered an Oxzilla FC-2 O_2_ analyzer (Sable Systems), after which air was ejected. Preliminary measurements were performed to ensure stability of flow rate through either channel by measuring the flow rate of air ejected from the O_2_ analyzer. After the measurement, pupae were weighed using a Mettler Toledo MT5 microbalance (Columbus, USA) and left at 25°C with 12:12 light:dark cycle until adult eclosion, at which point they were sexed.

Differential CO_2_ and O_2_ were calculated by subtracting the output of the reference line from the output of the sample line. For all measurements, sampling rate was 1 Hz. In the program Expedata (version 1.9.10), the raw output was baseline corrected against the reference line value, fractioned and multiplied with flow rate to yield CO_2_ and O_2_ in ml min^−1^ (46). The values were then corrected by subtracting the readings from the empty control syringe from the sample values. MR was calculated by first integrating the fractioned CO_2_ and O_2_ (ml min^−1^) values against time to yield CO_2_ and O_2_ in ml produced while pupae were in the syringes. Next, *V*CO_2_, and *V*O_2_ were corrected by accounting for the fraction of air that was still left in the syringe and the time spent in the syringe using the formula (only calculation for *V*CO_2_ is shown) *V*CO_2_ = (CO_2_ × (0.6/0.45))/hours in syringe (46). Then, the RQ was calculated as RQ = *V*CO_2_/*V*O_2_. RQ values provide an estimate on what energy source is being catabolized to fuel metabolism (47). MR (in watts = joules s^−1^) was converted from *V*O_2_ using the formula MR = (*V*O_2_ × (16 + (5.164 × RQ)))/(60 ×60) (46) and finally divided by body weight in mg to yield MR mg^−1^.

In the present study, we monitored single identified individuals throughout pupal development, and sexed them after eclosion. For the vast majority, eclosion was successful and therefore we could use the true weight of the individual for the calculation above. However, for individuals that failed to eclose properly, we instead used the average weight for that sex and treatment to calculate MR.

### Statistical Analysis

All results are presented as means ± SEM. We first investigated normality of data using Shapiro–Wilk's normality test and then used one-way analysis of variance (ANOVA) or Student's *t*-test, followed by Tukey's multiple comparisons test. Lifespan data were subjected to survival analysis (Log rank tests with Mantel-Cox post-test) and presented as survival curves.

For the respirometry data, we used the natural logarithm of MR mg^−1^ due to deviations from normality. A factorial two-way ANOVA was used with MR mg^−1^ or RQ as dependent variable, and sex and treatment as factorial explanatory variables. Non-significant interactions and main effects were removed from final models (48). The respirometry data were analyzed with the IBM SPSS statistics 23.0 (IBM SPSS Inc., Chicago, IL, USA) statistical software package. Prism GraphPad version 6.00 (La Jolla, CA, USA) was used for generating all the graphs.

## Results

### Mutation of *dilp1* Decreases Body Weight

**Figure 1:**
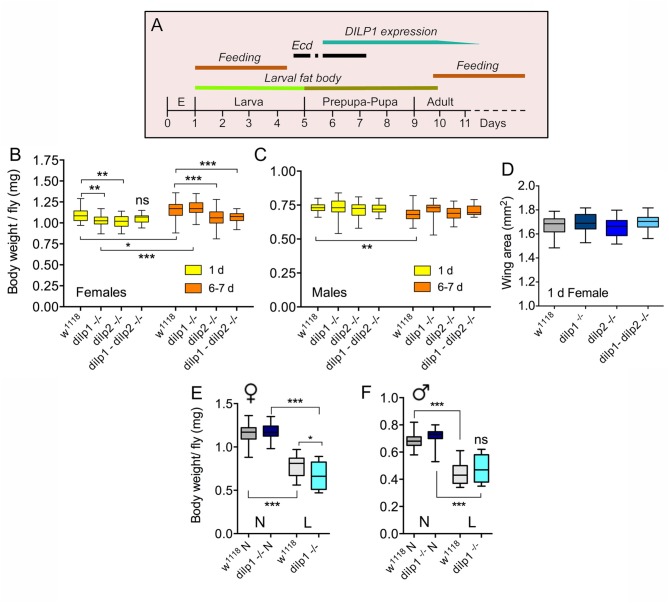
*dilp1* mutant flies display reduced body weight, but are not smaller. **(A)** Expression profile of *dilp1*/DILP1 in *Drosophila*. Note that expression of transcript and peptide coincides with the non-feeding pupal stage and the first days of adult life when food intake is reduced (especially day one). It also times with the onset of the second and third ecdysone (*Ecd*) surges in the early pupa (earlier ecdysone peaks are not shown). E, embryo. **(B)** Body weight of female flies 1 day and 6–7 days after adult eclosion. *dilp1* mutant flies display reduced body weight when 1 day old, but gain substantially the first week. Also, dilp2 mutants weigh less, but do not gain much weight the first week. The double mutants are not significantly affected compared to controls at 1 day, but after 6–7 days, both *dilp2* and double mutants weigh less that controls and *dilp1* mutants. Data are presented as medians ± range, *n* = 25–30 flies for each genotype from three independent replicates (*p < 0.05, **p < 0.01, ***p < 0.001, ns, not significant; two-way ANOVA followed by Tukey's test). **(C)** In male flies, the three mutants display weights similar to controls and controls lose weight the first week. Data are presented as medians ± range, *n* = 18–30 flies for each genotype from three independent replicates (**p < 0.01, two-way ANOVA followed with Tukey's test). **(D)** Wing area was used as a proxy for organismal growth. The three mutants did not display altered wing size. Data are presented as medians ± range, *n* = 16–23 flies for each genotype from three independent replicates (one-way ANOVA followed with Tukey's test). **(E,F)** Body weight of 7-day-old flies that had been exposed to normal diet (N) or low protein diet (L) during late larval stage. The female *dilp1* mutant flies displayed lower body weight than controls after low protein. Data are presented as medians ± range, *n* = 17–29 flies for each genotype from three replicates (*p < 0.05, ***p < 0.001, one-way ANOVA followed by Tukey's test).

It was previously reported that decreased *dilp1* activity reduces adult body weight in *Drosophila*, but it was not investigated at what developmental stage this occurred or whether the weight decrease was caused by diminished organismal growth (11, 20). This is relevant to ask since *dilp1* displays a restricted temporal expression during the *Drosophila* life cycle (see Figure 1A) and the body mass can increase without cellular/organismal growth. To analyze growth and other effects of *dilp1* and possible interactions with its tandem-encoded paralog *dilp2*, we employed recently generated *dilp1, dilp2*, and double *dilp1-dilp2* null mutants (35). The efficacy of these mutants was confirmed by qPCR in stage 8–9 pupae (about 50% pupal development) and immunolabeling in 1-week-old mated female flies (Supplementary Figure 1). We also asked whether the *dilp1* mutants displayed compensatory changes of other *dilps* in the IPCs or fat body. In *dilp1* mutant pupae (stage 8–9), the mRNA levels of *dilp2, dilp3*, and *dilp6* were not altered, but in *dilp6* mutants, the *dilp1* level was upregulated (Supplementary Figures 1A–C). These findings suggest that only minor (or no) compensatory changes in transcripts of other *dilps* in *dilp1* mutants occur during the mid-pupal stage (later pupal stages were not tested). In 1-week-old female flies, however, immunocytochemistry shows that at the protein level DILP2, but not DILP3, immunofluorescence increased in *dilp1* mutants (Supplementary Figures 1D–G). An earlier study showed upregulation also of *dilp2* transcript in flies of the same age (35). It should also be noted that the relative expression of *dilp1* is 100-fold lower than that of *dilp2* (35). Taken together, this suggests that *dilp2/*DILP2 could provide some compensation for lack of *dilp1* at least in young adult flies.

To determine a possible role of *dilp1* and *dilp2* in organismal growth during development, we initially monitored the body weight (wet weight) of *dilp1, dilp2*, and *dilp1/dilp2* double mutants. First, we measured the body weight in both recently eclosed and, for comparison, 6- to 7-day-old adult mated *dilp1* mutant flies. In female flies, the newly eclosed *dilp1* mutants displayed a decrease in body weight compared to controls (Figure 1B). However, this difference in body weight was no longer detectable in 6- to 7-day-old mated flies kept under normal feeding conditions; a significant weight increase was observed in both controls (*w*^1118^) and *dilp1* mutant flies, but not in *dilp2* and double mutants (Figure 1B). Also, *dilp2* mutant female flies have significantly lower body weight than controls 1 day after emergence, but in contrast to *dilp1* mutants, they did not increase the weight over 6–7 days of feeding (Figure 1B), possibly indicating that *dilp2* affects egg development. We will get back to these effects on “older” flies in a later section. Interestingly, the weight of *dilp1/dilp2* double mutants was not significantly affected compared to the single mutants (and control) and no weight increase was seen the first week, except in control flies (Figure 1B). Thus, there was no additive effect of the two mutations in females. In male flies, none of the mutant flies displayed altered body weight (Figure 1C). The effects of different genotypes on flyweight are shown in Table 1.

**Table 1:** Body weights of flies of different genotypes.

**Genotype**	**Wet weight females**		**Wet weight males**	
	**1-day adults**	**6- to 7-day adults**	**1-day adults**	**6- to 7-day adults**
*w^1118^*	1.094 ± 0.014	1.157 ± 0.021	0.733 ± 0.006	0.686 ± 0.009
*dilp1^−/−^*	1.024 ± 0.015^**^	1.180 ± 0.015	0.733 ± 0.12	0.718 ± 0.10
*dilp2^−/−^*	1.011 ± 0.015^**^	1.061 ± 0.019^***^	0.716 ± 0.10	0.691 ± 0.10
*dilp1/dilp2^−/−^*	1.055 ± 0.010	1.068 ± 0.012^***^	0.728 ± 0.007	0.710 ± 0.009
*dilp2>dilp1-Ri*	0.935 ± 0.014^*^	nt	0.721 ± 0.012	nt
*dilp2>w^1118^*	1.055 ± 0.013	nt	0.722 ± 0.001	nt
*dilp2>w^1118^*	1.055 ± 0.013	1.097 ± 0.018	0.722 ± 0.010	0.674 ± 0.007
*w^1118^>dilp1*	1.019 ± 0.025	0.984 ± 0.044	0.721 ± 0.011	0.676 ± 0.011
*dilp2>dilp1*	1.065 ± 0.025	1.201 ± 0.023^**^	0.760 ± 0.010^*^	0.716 ± 0.008^*^
*ppl>w^1118^*	0.903 ± 0.023	1.216 ± 0.027	0.662 ± 0.016	0.644 ± 0.016
*w^1118^>dilp1*	0.996 ± 0.030	1.155 ± 0.027	0.721 ± 0.011	0.650 ± 0.011
*ppl>dilp1*	1.096 ± 0.029^*^	1.340 ± 0.038^*^	0.876 ± 0.031^***^	0.743 ± 0.021^**^
*to>w^1118^*	0.909 ± 0.019	1.189 ± 0.018	0.667 ± 0.014	0.623 ± 0.011
*w^1118^>dilp1*	0.925 ± 0.019	1.192 ± 0.020	0.651 ± 0.011	0.674 ± 0.008
*to>dilp1*	1.055 ± 0.019^**^	1.295 ± 0.029^**^	0.753 ± 0.016^***^	0.706 ± 0.006^***^

Wet weights are given in mg ± SEM

(* p < 0.01,

** p < 0.01, and

*** *p < 0.001 compared to controls; one-way ANOVA was used for comparing three groups or more, unpaired Student's t-test was used for pairwise comparisons; see figure legends for further data). nt, not tested*.

To determine whether decreased organismal growth was responsible for the lower body weight, we measured wing size in 1-day-old female mutant flies and found no significant difference to controls (Figure 1D). Thus, the decreased weight of the flies does not seem to reflect a significant decrease in organismal size. We cannot exclude that the lack of a growth phenotype in the dilp1 mutants is caused by compensatory action of other DILPs. It was shown in a previous study that 1-week-old *dilp1* mutant flies display a 2-fold increased expression of *dilp6* transcript (17) that might compensate for the loss of *dilp1*.

What is the role of *dilp1* during pupal development? In a study of *dilp6*, it was shown that if third instar larvae (after reaching critical size) were put on a low-protein diet, they emerged as adults with lower body mass (wet weight) and that this was accentuated in *dilp6* mutants (16). This suggests that *dilp6* is important for metabolism and to assure growth of adult tissues under low protein conditions. We, thus, performed a similar experiment with *dilp1* mutant larvae kept on normal food or low protein diet. Flies emerging from larvae on restricted protein indeed displayed significantly lower body weight and female *dilp1* mutants weighed less than controls under protein starvation (Figure 1E). In male flies, this latter effect was not seen in the mutants (Figure 1F). This sex difference might indicate that part of the female weight loss is caused by diminished egg or ovary development.

We then asked whether mutation of both *dilp1* and *dilp6* would result in a further decrease of body weight and generated a recombinant *dilp1/dilp6* mutant. Using qPCR, we found that these flies displayed virtually no detectable *dilp1* and *dilp6* RNA (Supplementary Figure 2A). The weights of *dilp1/dilp6* mutants were significantly reduced compared to controls (Supplementary Figure 2B). However, their weights were not diminished more than those of the single *dilp1* and *dilp6* mutants, suggesting that there was no additive effect caused by loss of both *dilps*.

### Overexpression of *dilp1* Promotes Organismal Growth

Having shown effects of the *dilp1* null mutation on adult fly weight, we next explored the outcome of overexpressing *dilp1*, either in IPCs, or more broadly, in fat body, or using a neuroendocrine cell Gal4 driver, c929. The fat body expression represents fully ectopic *dilp1* expression (gain of function) since we could not detect *dilp1/*DILP1 in the fat body at any stage in wild-type flies; we rely here on the capacity of the fat body to produce and release DILP1 similar to DILP6. For the overexpression, we used several UAS-*dilp1* lines [see Post et al. (35)]. These UAS-*dilp1* lines were verified by DILP1 immunolabeling after expression with several Gal4 drivers (Supplementary Figures 3A–D) and by qPCR in stage 8–9 pupae (Supplementary Figures 4A–F). Overexpression of *dilp1* in the fat body, using the fat body-specific *pumpless (ppl)* and *takeout (to)* Gal4 drivers, and in IPCs (*dilp2*-Gal4) results in a drastic upregulation of *dilp1* RNA (Supplementary Figures 4A,D), but has no effect on *dilp2* and *dilp6* expression (Supplementary Figures 4B,C,E,F), except a minor decrease in *dilp2* for *ppl*-Gal4 (Supplementary Figure 4B). At the protein level, *dilp1* overexpression resulted in upregulation of DILP2 and DILP5 immunolevels in IPCs and a minor downregulation of DILP3 of 1-week-old adult female flies (Supplementary Figures 5A–E). One line, UAS-*dilp1* (III), was selected for subsequent experiments since it generated the strongest DILP1 immunolabeling.

To determine whether manipulations of *dilp1* affect the body mass during pupal development, we monitored wet weights of flies on the first day after eclosion (1-day-old flies). First, we used a *dilp2*-Gal4 driver to express *dilp1* in the IPCs and detected no significant increase in body weight of female flies (Supplementary Figure 2A; Table 1), but a slight increase in males (Supplementary Figure 2B; Table 1). We next expressed *dilp1* in the fat body, the insect functional analog of the liver and white adipocytes in mammals, and the source of secreted DILP6 (16, 31, 49–51). The fat body displays nutrient sensing capacity and is an important tissue for regulation of growth and metabolism in *Drosophila* either by secreted DILP6 or via other factors acting on IPCs to affect DILP secretion (16, 31, 51–55). To investigate the effect of ectopic *dilp1* expression in the fat body, we used the *ppl* and *to* Gal4 drivers. The efficiency of the drivers was confirmed by DILP1 immunostaining of larval fat body of *ppl*>*dilp1* and *to*>*dilp1* flies, but not in the control flies (Supplementary Figure 3D). In *ppl*>*dilp1* flies, we also found DILP1 labeling in the nephrocytes (not shown), which are highly endocytotic cells located close to the heart (56). The immunoreactive DILP1 is likely to have accumulated from the circulation after release from the fat body since the *ppl*-Gal4 is not expressed in the nephrocytes.

**Figure 2:**
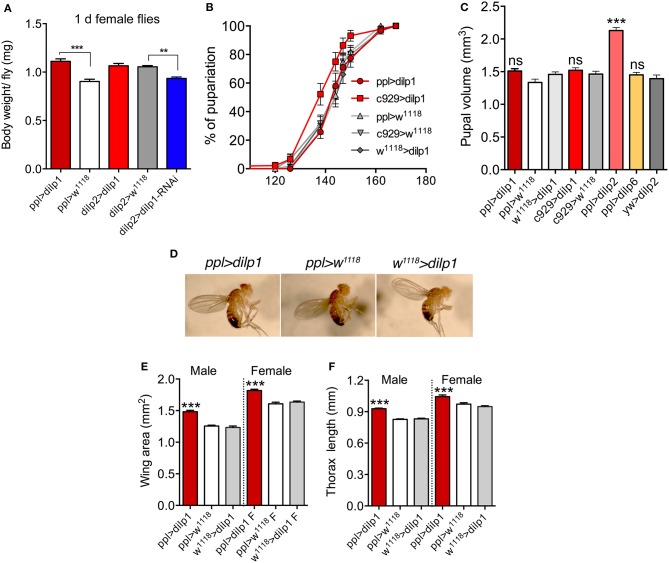
Overexpression of *dilp1* affects growth during pupal stage. **(A)** One-day-old female flies weigh more than controls in *ppl*>*dilp1* flies, but not in *dilp2*>*dilp1*. Knockdown of *dilp1* by *dilp2*>*dilp1*-RNAi leads to decreased body weight. Data are presented as means ± S.E.M, *n* = 20–27 flies for each genotype from three independent replicates (**p < 0.01, ***p < 0.001, unpaired Student's *t*-test). **(B)** Overexpression of *dilp1* in fat body (*ppl*-Gal4) or neuroendocrine cells (*c929*-Gal4) does not affect time to pupariation (larval development). Data are presented as means ± S.E.M, *n* = 138–147 flies for each genotype from three independent replicates [assessed by Log-rank (Mantel-Cox) test]. **(C)** Overexpression of *dilp1* using *ppl*-Gal4 or *c929*-Gal4 does not affect pupal volume (proxy for larval growth). Also *dilp6* overexpression has no effect, whereas *dilp2* expression triggers a significant increase in pupal volume. Data are presented as means ± S.E.M, *n* = 15–32 flies for each genotype from three independent replicates (***p < 0.001, ns, not significant; one-way ANOVA followed with Tukey's test). **(D)** Images of flies overexpressing *dilp1* in the fat body and controls. **(E,F)** Overexpression of *dilp1* in fat body results in flies with increased wing area **(E)**, and length of thorax **(F)** as proxies for organismal growth. Data are presented as means ± S.E.M (***p < 0.001, one way ANOVA followed with Tukey's test); in H *n* = 17–24 flies and in I *n* = 9–17 flies from three independent replicates.

We found that ectopic expression of *dilp1* in the fat body (*ppl*>*dilp1*), but not in IPCs (*dilp2*>*dilp1*), increased the body weight of females (Figure 2A; Table 1) as well as males (Supplementary Figure 6C; Table 1). We suggest that *dilp1* overexpression in IPCs, which already express the peptide, does not necessarily lead to increased release of DILP1; the amount of release is likely to be tightly controlled and is not affected by the size of the stored pool of peptide. In contrast, knockdown of *dilp1* in IPCs (*dilp2*>*dilp1*-RNAi), leads to a decrease in body weight (Figure 2A).

Before monitoring further effects of *dilp1* overexpression in the fat body on regulation of adult body weight and organismal size, we wanted to determine whether *dilp1* has an effect on larval development and/or growth. We therefore measured the time from egg to pupariation and size of pupae to determine whether *dilp1* overexpression affected timing of larval development and growth during this stage. Using the *ppl*-Gal4 driver, we did not observe any effect on the time from egg to pupa compared to controls (Figure 2B). Pupal volume, as a measurement of larval growth, was not altered by *ppl*-Gal4>*dilp1* (Figure 2C), suggesting that the larval growth was not affected. As expected (16, 31), overexpression of *dilp6* also had no effect on pupal size (Figure 2C). However, as shown earlier for ubiquitously expressed *dilp2* (23), *dilp2* expression in the fat body generated a strong increase in pupal volume, suggesting that growth occurred during the larval stage (Figure 2C). Driving *dilp1* with the *c929* Gal4 line, which directs expression to several hundred *dimm*-expressing peptidergic neurons including IPCs and other neurosecretory cells (57), we also did not observe any effect on time to pupariation or pupal volume (Figures 2B,C). Taken together, our data suggest the ectopic *dilp1* does not affect larval growth or developmental time, whereas dilp2, as shown earlier, does affect larval growth (developmental time was not monitored). Since only one receptor (dInR) is known for DILP1, DILP2, and DILP6 in *Drosophila*, the differential action of these peptides on larval growth could perhaps be explained by stage-specific and differential control by insulin/IGF-binding proteins such as secreted decoy of insulin receptor (SDR), acid-labile subunit (ALS), and imaginal morphogenesis protein-Late 2 (Imp-L2), which are known to inhibit the action of DILPs and affect growth (55, 58–61).

Next, we found that organismal size, estimated by wing size (Figures 2D,E) and thorax length (Figures 2D,F), increased after ectopic expression of *dilp1* in the fat body. Thus, overexpression of *dilp1* does not just lead to a weight increase, but to actual growth of the organism. Since we see no effect of *dilp1* expression on developmental time or pupal volume, but register increased body weight and size of adults, we propose that ectopic *dilp1*, like *dilp6*, promotes growth of adult tissues during the pupal stage. This stage also correlates with the temporal expression pattern of dilp1/DILP1 (17).

To substantiate the data obtained with the *dilp2* and *ppl*-Gal4 drivers, we also tested ectopic *dilp1* expression using another fat body driver, *to*-Gal4, and a neuroendocrine cell driver, c929. With *to*-Gal4>*dilp1*, we also noted an increase in weight of recently emerged female and male flies (Supplementary Figures 6D,E; Table 1), but no change in body size except a minor increase in thorax length in females (Supplementary Figures 6F,G). The female *to*>*dilp1* flies increased further in weight the first 6–7 days of adult life, but not later (Supplementary Figure 6D), whereas the males did not (Supplementary Figure 6E). Furthermore, with the *to*-Gal4 driver, there was no increase in pupal volume, supporting that *dilp1* does not affect larval growth (Supplementary Figure 6H).

Ectopic expression of *dilp1* in neuroendocrine cells by means of the *c929*-Gal4 increased adult body weight (Supplementary Figure 7A), but had no effect on wing area in males and females (Supplementary Figure 7B), suggesting that the *dilp1* expression (and/or systemic release) was not strong enough to produce major effects. As mentioned, expressing *dilp1* in cells already producing it (IPCs) may not yield increased release and an ensuing phenotype, and additional neurosecretory cells in the c929 line may not release enough DILP1.

### MR and RQ in Pupae of Different Genotypes

**Figure 3:**
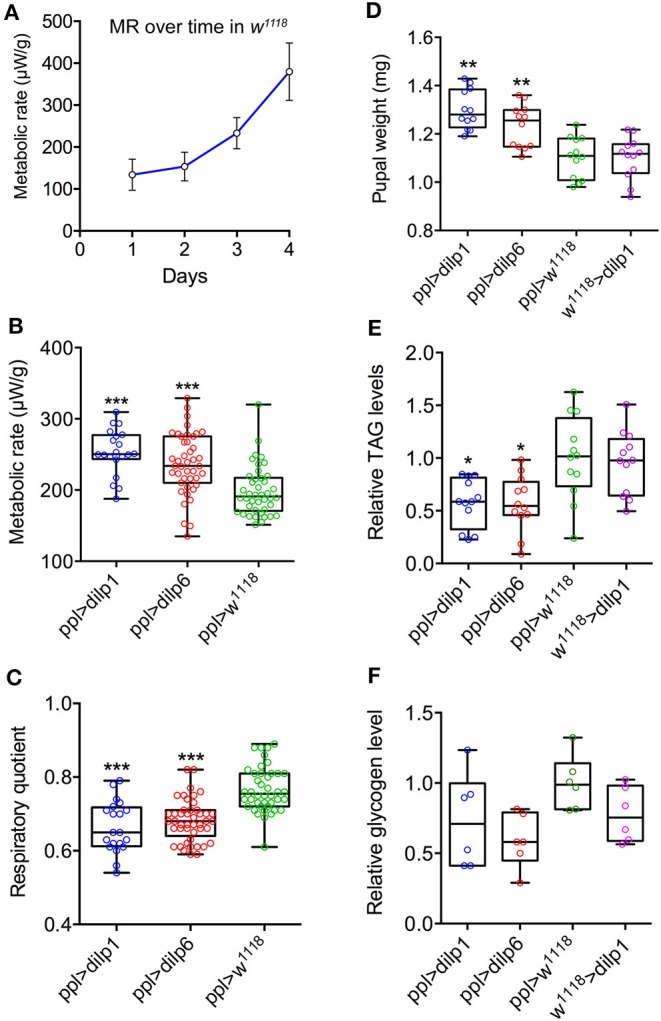
Metabolic rate trajectories and respiratory quotients (RQs) during pupal development respond to *dilp1* and *dilp6* overexpression in the fat body. **(A)** Metabolic rate in *w*^1118^ flies increased exponentially as a function of time. For the ensuing overexpression analysis, we studied the period 96–120 h after pupation. Data are presented as means ± S.E.M, *n* = 20–47 flies from three independent replicates. **(B)** Metabolic rate was significantly elevated during this period in *dilp1* and *dilp6* overexpression flies (*ppl-Gal4*) when compared to *w*^1118^ flies. Data are presented as means ± S.E.M, *n* = 20–47 flies for each genotype from three independent replicates (***p < 0.001, compared to *w*^1118^ flies, as assessed by two-way ANOVA followed with Tukey's test). Data are from both males and females as no difference was found in the ANOVA for sex. **(C)** RQ, reflecting catabolic energy substrate, was significantly lower in the overexpression flies when compared to the control flies and indicates a shift from mixed fuel catabolism (RQ = 0.7–0.8) to predominantly lipid catabolism (RQ < 0.7). Data are presented as means ± S.E.M, *n* = 20–47 flies for each genotype from three independent replicates (***p < 0.001, compared to *w*^1118^ flies, as assessed by one-way ANOVA followed with Tukey's test). Data are from both males and females as no difference was found in the ANOVA for sex. **(D)** Four-day-old pupae (mixed male and female) were weighed (wet weight) before extraction and TAG determination. Overexpression of *dilp1* and *dilp6* both resulted in increased pupal weight. **(E)** Levels of TAG were measured in the pupae used for weighing in D. Overexpression of each *dilp* resulted in decreased TAG levels. **(F)** Glycogen levels in 4-day-old pupae (no significant changes). In **(D,E)**, 12 replicates per genotype with 4 pupae in each replicate (each data point represents 4 pupae); in **(F)**, 6 replicates per genotype with 4 pupae in each replicate (*p < 0.05, **p < 0.01, one-way ANOVA followed by Tukey's test).

To investigate a possible role of *dilp1* in metabolism and utilization of nutrients during pupal development, metamorphosis, and adult tissue growth, we determined MR and RQ in pupae of different genotypes. First we characterized the metabolic trajectory in control pupae (*w*^1118^) by measuring cumulative MR daily throughout pupal development (Figure 3A). These data show the exponential MR curve typical for developing insects, including *D. melanogaster* (62). To minimize handling stress, we chose to investigate only the end of pupal development in more detail and measured MR and RQ in 4-day-old pupae (that is the cumulative MR between hours 96 and 120 after pupation). For this experiment, we only obtained useful data for *ppl*-Ga4 overexpression animals since the mutant flies displayed high mortality in the respirometry setup and therefore numbers of data points obtained were small (not shown). Instead, we monitored the effect of *dilp1* gain of function by expression in the fat body using *ppl*-Gal4 (to match other gain of function experiments). As can be seen in Figures 3B,C, the *ppl*>*dilp1* and *ppl*>*dilp6* differed significantly from the controls in the respirometry assay. The MR was higher and RQ was lower in the *dilp1* and *dilp6* overexpression flies than in the control flies. RQ values, around 0.6 in both overexpression lines, suggest pure lipid metabolism (47), and lipids are known to be a major or sole fuel during metamorphosis of insects (45, 62–64). Our findings strongly suggest that *dilp1* and *dilp6* affect metabolism (especially of lipids) in the pupa, probably by acting on the residual larval fat body that is present throughout pupal development and the first few days of adult life (65, 66).

### TAG and Carbohydrates in Pupae of Different Genotypes

To determine whether lipids indeed fuel growth of adult tissues in 4-day-old pupae, we determined TAG levels after overexpression of *dilp1* and *dilp6* in fat body (*ppl*-Gal4). Pupae of both genotypes displayed increased weight (Figure 3D) and also significantly reduced TAG levels (Figure 3E), compared to controls of the same age. The levels of glycogen were not significantly altered in pupae after ectopic expression of *dilp1* and *dilp6* (Figure 3F) and neither were glucose levels (Supplementary Figure 8).

### Effects of *dilp1* Manipulations on Metabolism and Body Mass in Newly Eclosed and Young Flies

**Figure 4:**
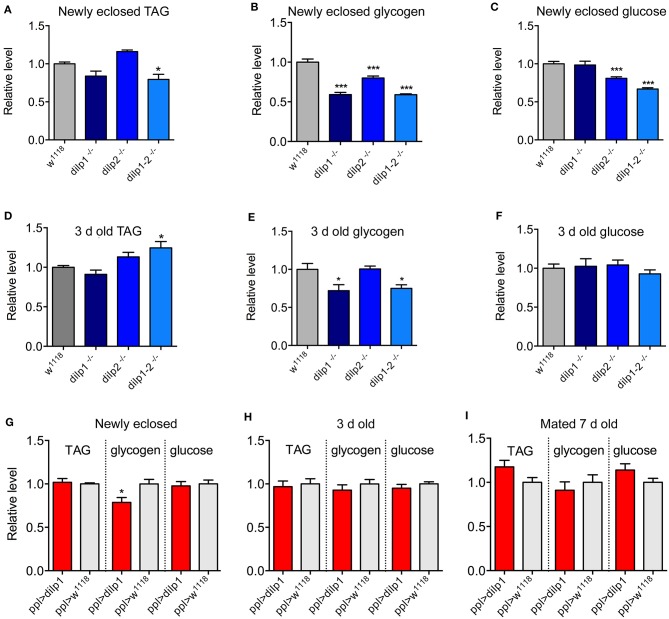
Contents of TAG, glycogen, and glucose in female mutant flies and after ectopic *dilp1* expression. **(A–C)** Contents of TAG and carbohydrates in recently emerged mutants and controls. Note that for *dilp1* mutants, only glycogen was diminished, whereas for *dilp1-2* mutants, all three compounds were decreased. Eight replicates per genotype with 5–6 flies in each replicate (*p < 0.05, ***p < 0.001, one-way ANOVA followed by Tukey's test). **(D–F)** In 3-day-old flies, glycogen was also reduced in *dilp1* mutants and double mutants. Eight replicates per genotype with 5–6 flies in each replicate (*p < 0.05, ***p < 0.001, one-way ANOVA followed by Tukey's test). **(G–I)** Overexpression of *dilp1* in fat body (*ppl-Gal4*) only affected glycogen levels in newly emerged flies. Six to eight replicates per genotype with 5–6 flies in each. Data are presented as means ± S.E.M (*p < 0.05, ***p < 0.001, compared to *w*^1118^ flies, as assessed by unpaired Student's *t*-test).

We have shown that *dilp1*/DILP1 expression is prominent in pupae as well as during the first 5–7 days of adulthood (17). What is the role of the peptide in young flies? To investigate whether *dilp1*/dilp1 signaling affects adult metabolism, we monitored the levels of TAGs, glycogen, and glucose in recently emerged and 3-day-old *dilp* mutant and *dilp1*-overexpressing female flies (Figure 4). In newly eclosed *dilp1* mutant flies, glycogen was significantly lowered, whereas glucose and glycogen were diminished in *dilp2* mutants, while in the *dilp1/dilp2* double mutants, all three compounds were decreased (Figures 4A–C). In the 3-day-old flies, *dilp1* and double mutants displayed reduced glycogen, whereas in *dilp1/dilp2* double mutants, TAG was increased (Figures 4D–F).

Using *ppl*-Ga4 to overexpress *dilp1*, we found that the only effect was a reduction of glycogen in recently eclosed flies; at 3 or 7 days of age, no effect was noted (Figures 4G–I). Thus, it appears that intact *dilp1* signaling is required for mobilization of glycogen stores in newly emerged and young flies. The finding that both *dilp1* mutants and flies with *dilp1* overexpression display decreased glycogen may suggest that any change of *dilp* signaling offsets glycogen homeostasis.

**Figure 5:**
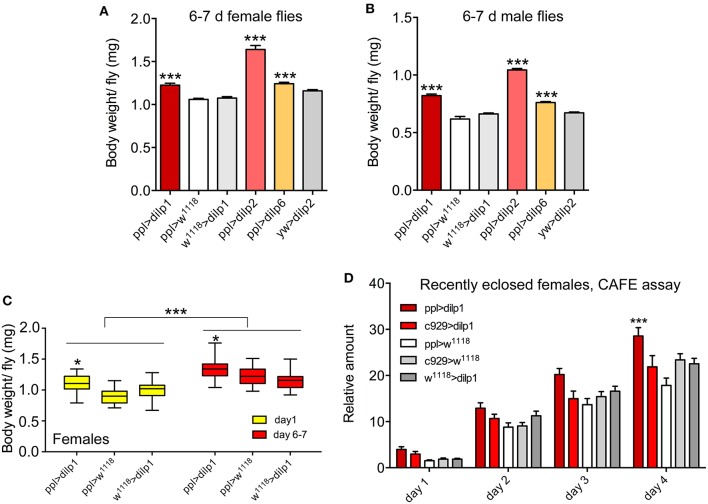
Overexpression of *dilp1* affects body mass and feeding in young adult flies. **(A**, **B)** Overexpression of *dilp1, dilp2*, and *dilp6* in fat body (*ppl*-Gal4) all lead to adult flies (1 week old) with increased body weight in both females and males. Data are presented as means ± S.E.M, *n* = 24–30 flies for each genotype from three independent replicates. Except for ppl>dilp2, 13 flies were used (*p < 0.05, one-way ANOVA followed with Tukey's test). **(C)** Body weight of 6- to 7-day female flies is increased for all genotypes compared to 1-day flies and the *ppl*>*dilp1* flies weigh more than controls at both time points. Data are presented as medians ± range, *n* = 23–27 flies for each genotype from three independent replicates (*p < 0.05, ***p < 0.001, two-way ANOVA followed with Tukey's test). **(D)** Food intake (CAFE assay) is increased over 4 days (cumulative data shown) in flies overexpressing *dilp1* in fat body, but not in neuroendocrine cells (*c929*-Gal4). Data are presented as means ± S.E.M, *n* = 15–30 flies for each genotype from three independent replicates (*p < 0.05, two-way ANOVA followed with Tukey's test).

Next we asked whether dilp1 has an effect on body mass and food ingestion in adult flies. Hence, we first determined the wet weight of mated 6- to 7-day-old flies and found that it was significantly increased in *ppl*>*dilp1* flies compared to the controls in both female (Figure 5A; Table 1) and male flies (Figure 5B). We furthermore noted increased weight for *ppl*>*dilp2* and *ppl*>*dilp6* flies (Figures 5A,B; Table 1). Comparing 1-day-old female flies with 6- to 7-day-old ones, it is clear that all genotypes display increased body mass, but at each time point, the *ppl*>*dilp1* flies increase significantly more than controls (Figure 5C). This weight increase in all genotypes of females may be related to ovary growth, since male flies do not increase their weight the first week; they instead weigh less (Supplementary Figure 6C). However, since male *ppl*>*dilp1* flies also display higher body mass than controls at both time points, it is suggestive that *dilp1* plays a role in regulation of other aspects of body mass.

As a comparison, *dilp2*>*dilp1* had only minor effects on body weight of female flies; only in 6- to 7-day-old *dilp2*>*dilp1* flies was there an increase compared to the controls of the same age (Supplementary Figure 6A; Table 1), whereas in males, a significant increase in weight was noted at both ages for *dilp2*>*dilp1* compared to controls, and a loss of weight over the next 6 days for all genotypes (Supplementary Figure 6B). Using another fat body Gal4-driver, *to-*Gal4, to express *dilp1*, we obtained results similar to *ppl*-Gal4 (Supplementary Figures 6D,E). Monitoring body mass another week (13–14 days) in *to*>*dilp1* flies, we found that, in males, *dilp1*-overexpressing flies are still heavier than controls, whereas in females, the different genotypes weigh the same (Supplementary Figures 6D,E). Thus, these experiments indicate that there is a sex difference in the body mass profile over the first week that might reflect egg development. However, *dilp1* overexpression leads to increased body mass in both sexes compared to controls in both 1-day and 6- to 7-day-old flies.

It was suggested that *dilp6* promotes growth of adult tissues during pupal development by utilizing nutrients stored in the larval fat body, which is carried into the pupa (16). This may be the case also for *dilp1*, and if so, newly eclosed *dilp1*-overexpressing flies should have less energy stores in the residual larval fat body. Also, the increased body mass over the first week requires additional nutrients. To test this, we monitored feeding in recently emerged *dilp1*-overexpressing flies (*ppl*>*dilp1*) and controls. Indeed, flies overexpressing *dilp1* displayed increased food ingestion over the first 4 days after adult emergence compared to controls (Figure 5D), suggesting that these flies were in extra need of nutrients.

### Effects of *dilp1* on Adult Stress Resistance and Fecundity

**Figure 6:**
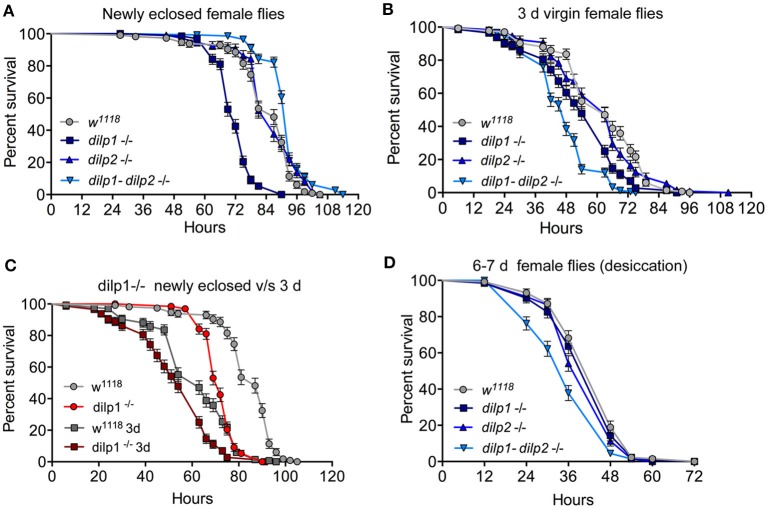
Effects of mutated *dilp* genes on adult responses to starvation and desiccation change in early adult life. **(A)** In newly eclosed female flies, *dilp1* mutant flies display reduced survival during starvation (*p* < 0.001) compared to the other mutants and control. The double mutant is significantly more resistant (*p* < 0.001). *n* = 109–147 flies for each genotype from three independent replicates. **(B)** In 3-day-old virgin female flies, *dilp1-dilp2* double mutants are the least starvation resistant (*p* < 0.001) followed by the *dilp1* mutants; *n* = 129–148 flies for each genotype from three independent replicates. **(C)** Comparison between newly eclosed and 3-day-old flies exposed to starvation. Both mutants and controls survive longer as recently eclosed flies and mutants perform worse than controls at each time point (*p* < 0.001). *n* = 114–144 flies from three independent replicates. **(D)** When exposed to desiccation, 6- to 7-day-old female double mutants are less resistant than the other genotypes (*p* < 0.001), *n* = 132–135 flies from three independent replicates. Data are presented in survival curves and the error bars show S.E.M., as assessed by log-rank (Mantel–Cox) test.

Genetic ablation of the IPCs, which produce DILP1, 2, 3, and 5, results in increased starvation resistance in adult flies (21). Thus, we asked whether alterations of *dilp1* expression have effects on adult physiology such as survival during starvation or desiccation (also referred to as starvation and desiccation resistance). We investigated the starvation resistance in newly emerged, 3-day-old and 1-week-old female *dilp1, dilp2*, and *dilp1/dilp2* mutant flies (all virgins). The newly eclosed *dilp1* mutant flies display strongly reduced survival during starvation and the *dilp1/dilp2* mutants increased survival compared to control flies, whereas the starvation resistance of *dilp2* mutants is similar to the controls (Figure 6A; Table 2). In 3-day-old virgin flies, the *dilp1* and *dilp1/dilp2* mutants display reduced survival during starvation, whereas the *dilp2* mutants perform similar to the controls (Figure 6B; Table 2). In a previous study (35), it was shown that 6- to 7-day-old female flies display a similar response to starvation: the *dilp1/dilp2* mutants exhibit the strongest reduction in survival, followed by *dilp1* mutants that also are much less stress tolerant, whereas the performance of *dilp2* mutants and control flies is very similar (see Table 2). Here, we also tested 6- to 7-day-old male flies and found that they survived starvation in a manner different from females with *dilp2* and double mutants displaying diminished stress resistance, whereas *dilp1* mutants survive similar to controls (Supplementary Figure 9A). Thus, a difference between sexes was detected in metabolic stress responses of the different mutants that might suggest a link between *dilp1* and egg development in females. We also see a change in the effects of *dilp1* mutation over age in female flies that may reflect the switch from larval to adult fat body, as well as ovary maturation.

**Table 2:** Median lifespans of *dilp1* mutant and *dilp1* overexpressing female and male flies exposed to starvation display a sex dimorphism.

**Genotype**	**Female median lifespan (calculated as % of w**^****1118****^**)**			**Males**
*w^1118^*	100	100	100	100
*dilp1^−/−^*	83 (*p* < 0.001)	86 (*p* < 0.001)	78 (*p* < 0.001)	125 (*p* < 0.001)
*dilp2^−/−^*	100	100	100	125 (*p* < 0.001)
*dilp1/dilp2^−/−^*	107 (*p* < 0.001)	76 (*p* < 0.001)	67 (*p* < 0.001)	100
*ppl>w^1118^*	100	–	100	100
*ppl>dilp1*	80 (*p* < 0.001)	–	90 (*p* < 0.001)	100

^*^Data from Post et al. (35).

As seen above, our data suggest a change in the response to loss of *dilp1* and *dilp1/dilp2* function in starvation resistance over the first week of adult life. It is known that newly eclosed wild-type flies are more resistant to starvation than slightly older flies (66). Thus, we compared the survival during starvation in recently emerged and 3-day-old virgin flies. As seen in Figure 6C (based on data in Figures 6A,B), recently eclosed control flies (*w*^1118^) indeed exhibit increased starvation resistance compared to controls that were tested when 3 days old. Also, the *dilp1* mutant flies are more starvation resistant when tested as newly eclosed than as older flies, and the mutants perform less well than controls at both ages (Figure 6D). However, the most drastic change within the first week is that *dilp1* mutants yield the strongest reduction in starvation resistance as newly eclosed flies, and then in 3-day and 6- to 7-day-old flies, the *dilp1/dilp2* mutants are the ones with the lowest stress resistance. Thus, a change in the role of *dilp1* seems to occur as the fly matures during the first few days of adult life. To provide additional evidence that *dilp1* impairs starvation resistance, we performed *dilp1*-RNAi using a *dilp2*-Gal4 driver. The efficiency of the *dilp2*>*dilp1*-RNAi was tested by qPCR (Supplementary Figure 10A) where a strong decrease in *dilp1*, but not *dilp2* or *dilp6*, was seen. The *dilp1*-RNAi in IPCs resulted in newly eclosed flies that displayed reduced survival during starvation (Supplementary Figure 10B), similar to *dilp1* mutant flies.

Next, we investigated the effect of the mutations on the flies' response to desiccation (dry starvation). One-week-old flies were put in empty vials and survival was recorded. Female *dilp1/dilp2* mutants were more sensitive to desiccation than controls and both of the single mutants (Figure 6D). In males, the double mutants also displayed higher mortality during desiccation, whereas the two single mutants were *more* resistant than controls (Supplementary Figure 9B). Thus, there is a sex dimorphism in how the different mutants respond to both desiccation and starvation, and in female *dilp1* mutants, desiccation resistance seems not to be affected, in contrast to starvation resistance. This difference in response to desiccation may contribute to the sex dimorphism in wet weight after manipulating *dilp1* signaling.

**Figure 7:**
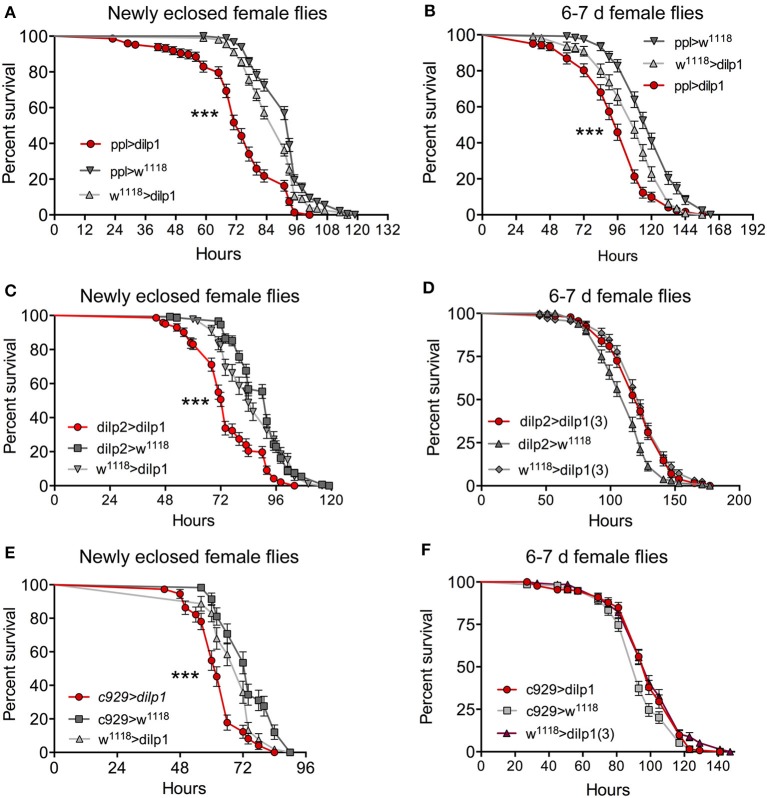
Overexpression of dilp1 in the fat body affects starvation resistance in adult flies. **(A**, **B)** In recently eclosed **(A)** and 6- to 7-day-old **(B)** female flies, overexpression of *dilp1* (with ppl-Gal4) leads to a decrease in survival during starvation *n* = 147–201 flies per genotype from three independent replicates [***p < 0.001, as assessed by log-rank (Mantel–Cox) test]. **(C,D)** Expressing *dilp1* in IPCs with a *dilp2*-Gal4 driver also diminishes starvation survival in recently eclosed flies, *n* = 92–148 flies from three independent replicates [***p < 0.001, as assessed by log-rank (Mantel–Cox) test], but not in 6- to 7-day flies (*n* = 122–132 flies from three independent replicates). **(E,F)** Using *c929* to drive *dilp1* in recently eclosed and 6- to 7-day-old adult flies altered starvation resistance only in the recently eclosed ones [***p < 0.001 as assessed by log-rank (Mantel–Cox) test, *n* = 132–135 flies per genotype from three independent replicates].

What about effects of *dilp1* gain of function on stress tolerance? When overexpressing *dilp1* with the fat body driver *ppl*-Gal4 newly eclosed and 6- to 7-day-old female flies become less resistant to starvation compared to parental controls (Figures 7A,B). However, in 6- to 7-day-old male flies, there is no difference between controls and flies with ectopic *dilp1*, using *ppl*- and *c929*-Gal4 drivers (Supplementary Figures 10C,D). We furthermore investigated starvation resistance in flies overexpressing *dilp1* in IPCs (*dilp2*>*dilp1*) and in most neuroendocrine cells (*c929*>*dilp1*) and found that, in just eclosed female flies, overexpression reduced survival (Figures 7C,E), whereas in 1-week-old flies, all genotypes displayed the same survival (Figures 7D,F). Thus, in females, it appears as if both knockout and overexpression of *dilp1* reduce starvation resistance in recently eclosed flies. It was shown earlier that conditional knockdown of *dilp6* by RNAi during the pupal stage resulted in newly eclosed flies with *increased* survival during starvation (16), suggesting that the effects of *dilp6* and *dilp1* mutation are different.

**Figure 8:**
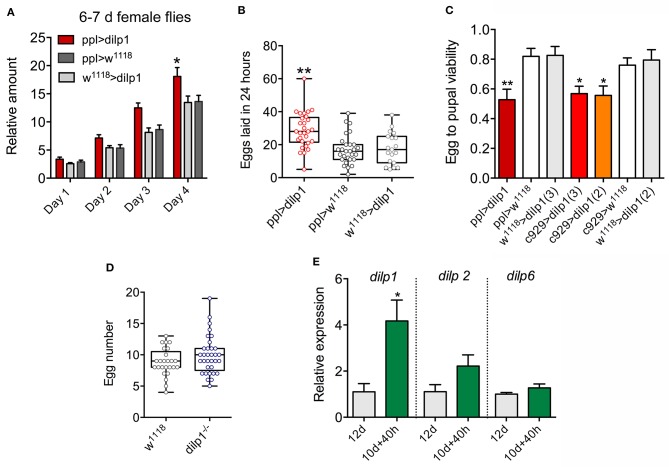
Overexpression of *dilp1* affects food intake and fecundity. **(A)** In CAFE assay, the *dilp1* overexpressing flies (6- to 7-day-old females) display increased food intake over 4 days (cumulative data shown). Data are presented as means ± S.E.M, *n* = 23–24 flies from three independent replicates (*p < 0.05, two-way ANOVA followed by Tukey's test). **(B)** Number of eggs laid in 24 h by 6- to 7-day-old flies. We analyzed 19–29 pairs of flies from 3 replicates (**p < 0.01, one-way ANOVA followed by Tukey's test). **(C)** The egg to pupal viability is diminished in flies with *dilp1* expressed in fat body (*ppl*-Gal4) and neuroendocrine cells [*c929*-Gal4, using two different UAS-*dilp1* (2 and 3)]. Data are presented as means ± S.E.M; more than 276 eggs from 6 replicates were monitored (*p < 0.05, unpaired Student's *t*-test). **(D)** Number of eggs in ovaries of 3-day-old flies is not affected in *dilp1* mutants. A total of 25–33 flies from three replicates were analyzed. **(E)** *dilp1* mRNA is upregulated during starvation for 40 h in 10-day-old adult *w*^1118^ flies, compared to 12-day-old flies fed normal food, as monitored by qPCR. No effect was seen on *dilp2* and *dilp6* levels. Data are presented as means ± S.E.M.; 3 replicates with 10 flies in each replicates were monitored (*p < 0.05, unpaired Student's *t*-test).

After ectopic expression of *dilp1* in the fat body, there was an increase in food intake (cumulative data) in 1-week-old flies over 4 days (Figure 8A), suggesting that metabolism is altered also in older flies. Since the effect of *dilp1* manipulations seems stronger in female flies, we asked whether fecundity is affected by overexpression of *dilp1*. An earlier study showed that *dilp1* mutant flies are not deficient in number of eggs laid, or the viability of offspring (egg to pupal viability), although the *dilp1/dilp2* double mutants displayed a reduction in viability of these eggs (35). Here, we expressed *dilp1* in fat body (*ppl*-Gal4) and detected an increase in number of eggs laid over 24 h in 6- to 7-day-old flies (Figure 8B). Both *ppl*-Gal4- and *c929*-Gal4-driven *dlip1* decreased the viability of eggs laid as monitored by numbers of eggs that developed into pupae (Figure 8C). As a comparison, we noted no difference in number of eggs retained in ovaries in 3-day-old *dilp1* mutant flies (Figure 8D) similar to the 6- to 7-day-old flies studied previously (35).

In flies older than 7 days kept under normal laboratory conditions, *dilp1*/DILP1 expression is barely detectable. Thus, we next asked whether there is any physiological trigger of increased *dilp1* expression in older adult flies, except for diapause (17) and experimental ones such as ectopic expression of sNPF or knockdown of *dilp6, dilp2* and *dilp2,3,5* (17, 35, 67). Here, we found that 40 h starvation of 10-day-old flies (*w*^1118^) leads to a significant increase in *dilp1*, but not in *dilp2* or *dilp6* (Figure 8E). Thus, at a time (12 days) when *dilp1* is barely detectable under normal conditions, there is a 4-fold upregulation during starvation, further suggesting that the peptide indeed plays a role also in older adult flies (and its function is uncoupled from its pupal role).

## Discussion

Our study shows that *dilp1* gain of function stimulates adult tissue growth and increases MR during the pupal stage, and also affects adult physiology, especially during the first days of adult life. These stages correspond to the time when *dilp1* is normally expressed (16, 17, 31). The gain of function experiments herein suggest that the developmental role of ectopic *dilp1* could be similar to that of *dilp6* (16), namely, to stimulate growth of adult tissues during pupal development. We furthermore show that in the adult fly, *dilp1* is upregulated during starvation and genetic gain and loss of function of *dilp1* signaling diminishes the flies' survival under starvation conditions in a sex-specific manner. These novel findings, combined with previous data that demonstrated high levels of *dilp1* during adult reproductive diapause (17) and the role of *dilp1* as a pro-longevity factor during aging (35), suggest a wide-ranging importance of this signaling system. Not only does *dilp1* expression correlate with stages of non-feeding (or reduced feeding), these stages are also associated with lack of reproductive activity and encompass the pupa, newly eclosed flies, and diapausing flies. Under normal conditions, the transient expression of *dilp1*/DILP1 during the first few days of adult life may be associated with a metabolic transition [remodeling from larval to adult fat body; (68)] and the process of sexual maturation (gonad growth and differentiation). Our data also suggest that *dilp1* affects physiology more prominently in young female flies than in males, which might be associated with ovary maturation.

It is also interesting to note that the diminished starvation resistance in *dilp1* and *dilp1/dilp2* mutants is opposite to the phenotype seen after IPC ablation, mutation of *dilp1-4*, or diminishing IIS by other genetic interventions (11, 21, 69, 70). Thus, in recently eclosed flies, *dilp1* appears to promote starvation resistance rather than diminishing it. Furthermore, the decreased survival during starvation in female *dilp1* mutants is the opposite of that shown in *dilp6* mutants (16), indicating that *dilp1* action is different from the other insulin-like peptides tested.

In *Drosophila*, the final body size is determined mainly by nutrient-dependent hormonal action during the larval feeding stage (10, 12, 23, 29). However, some regulation of adult body size can also occur after the cessation of the larval feeding stage, and this process is mediated by *dilp6* acting on adult tissue growth in the pupa in an ecdysone-dependent manner (16, 31). This is likely a mechanism to ensure growth of adult tissues if the larva is exposed to shortage of nutrition during its feeding stage. Our findings suggest that *dilp1* can function as another regulator of growth during the pupal stage. We show here that overexpression of *dilp1* promotes organismal growth in the pupa, probably at the cost of stored nutrients derived from the larval feeding stage. This is supported by our RQ data that clearly show a shift from mixed-energy substrate metabolism in control flies toward almost pure lipid catabolism at the end of pupal development in the *dilp1* overexpression flies (also seen for *dilp6* gain of function in our experiments). Furthermore, TAG (but not carbohydrate) levels in *dilp1* overexpression pupae were clearly decreased, which likely reflects the shift in catabolic energy substrate also seen in the RQ using respirometry. It should be noted that insects predominantly use lipids as fuel during metamorphosis (45, 62–64) and *dilp1* overexpression increases lipid catabolism. Our study hence suggests that *dilp1* can parallel *dilp6* (16, 31) in balancing adult tissue growth and storage of nutrient resources during pupal development. This is interesting since *dilp6* is an IGF-like peptide that is produced in the nutrient sensing fat body (16, 31), whereas the source of the insulin-like *dilp1* is the brain IPCs (17, 20).

In contrast to the *dilp1* gain of function, our experiments with *dilp1* mutant flies did not show a clear effect on adult body growth, only a decrease in weight. Is this a result of compensation by other DILPs? We showed earlier that young adult *dilp1* mutant flies display increased *dilp6* and vice versa (17), suggesting feedback between these two peptide hormones in adults. During the pupal stage, this feedback appears less prominent in *dilp1* mutants and we detected no effects on *dilp2, dilp3*, or *dilp6* levels. Furthermore, overexpression of *dilp1* in fat body or IPCs has no effect on pupal levels of *dilp2* and *dilp6*. Thus, at present, we have no evidence for compensatory changes in other *dilps*/DILPs in pupae with *dilp1* manipulations. However, under normal conditions (in wild-type pupae), *dilp6* levels are far higher than those of *dilp1* (31) [see also modENCODE_mRNA-Seq_tissues; (71)], which could buffer the effects of changes in *dilp1* signaling.

DILPs and IIS are involved in modulating responses to starvation, desiccation, and oxidative stress in *Drosophila* [see Grönke et al. (11), Broughton et al. (21), and Nässel and Vanden Broeck (55)]. Flies with ablated IPCs or genetically diminished IIS display increased resistance to several forms of stress, including starvation (11, 21). Conversely, overexpression of *dilp2* increases mortality in *Drosophila* (24). We found that young *dilp1* mutant flies displayed diminished starvation resistance. In both recently eclosed and 3-day-old flies, mutation of *dilp1* decreased survival during starvation (but not in 6- to 7-day-old ones).

Action of *dilp1* in the adult fly is also linked to reproductive diapause in females, where feeding is strongly reduced (72), and both peptide and transcript are upregulated (17). Related to this, we found here that *dilp1* mRNA is upregulated during starvation in 12-day-old flies. Furthermore, it was shown that expression of *dilp1* (*dilp1* rescue) increases lifespan in *dilp1/dilp2* double mutants, suggesting that loss of *dilp2* induces *dilp1* as a factor that promotes longevity (35). Thus, *dilp1* activity is beneficial also during adult life, even though its expression under normal conditions is very low (16, 17, 31). This pro-longevity effect of *dilp1* is in contrast to *dilp2, 3, and 5* and the mechanisms behind this effect are of great interest to unveil.

A previous study showed that in wild-type (Canton S) *Drosophila*, DILP1 expression in young adults is sex-dimorphic with higher levels in females (17). In line with this, we show here that starvation resistance in young flies is diminished only in female *dilp1* mutant and *dilp1* overexpression flies. Thus, taken together, we found earlier that *dilp1* displays a sex-specific expression (17) and here we show female-specific function in young adult *Drosophila*. It is tempting to speculate that the more prominent role of *dilp1* in female flies is linked to metabolism associated with reproductive physiology and early ovary maturation, which is also reflected in the *dilp1* upregulation during reproductive diapause (17). In fact, we show here that egg-laying increased after *dilp1* overexpression, and an earlier study demonstrated a decreased egg laying in *dilp1* mutant flies (17). Part of the sex dimorphic effects on body weight of young adults after dilp1 manipulations might be a result of a differential role of *dilp1* in water homeostasis.

We show here that IPC-derived *dilp1* displays several similarities to the fat body-produced *dilp6*, including temporal expression pattern, growth promotion, effects on adult stress resistance and lifespan. Additionally, *dilp1* may play a role in regulation of nutrient utilization and metabolism during the first few days of adult life, especially in females. At this time, larval fat body is still present and utilized as energy fuel/nutrient store (66) and this source also contributes to egg development (73). Curiously, there is a change in the action of DILP1 between the pupal and adult stages from being able to stimulate growth (agonist of dInR, like DILP6) in pupae, to acting in a manner opposite to DILP2, DILP6, and other DILPs in adults in regulation of lifespan and stress responses [see also Post et al. (35)]. Only one dInR is known so far (excluding the G protein-coupled receptors for the relaxin-like DILP7 and DILP8). Thus, the mechanisms behind this apparent switch in function of DILP1 signaling remain an open question. One possibility is that DILP1 acts via different signaling pathways downstream the dInR in pupae and adults. An obvious difference between these two stages is the presence of larval-derived fat body in the pupa and during the first few days of adults and its replacement by functional adult fat body in later stages (51, 66). Perhaps dInR-mediated action differs in these types of fat body when activated by DILP1. Another possibility is stage-specific expression of insulin/IGF-binding proteins such as SDR, ALS, and Imp-L2, mentioned earlier, that could affect the activity of DILP1 in particular [see Arquier et al. (58), Honegger et al. (59), and Okamoto et al. (60, 61)].

In the future, it would be interesting to investigate whether DILP1 acts differently on larval/pupal and adult fat body, or act on different downstream signaling in the two stages of the life cycle. Also, the possibility that *dilp1* and *dilp6* interact to regulate growth and metabolism in *Drosophila* is worth pursuing.

## Data Availability Statement

All datasets generated for this study are included in the article/Supplementary Material.

## Author Contributions

SL: conceptualization, performed experiments, interpreted data, and wrote paper. SP and PL: performed experiments and interpreted data. JV: contributed unpublished reagents. MT: conceptualization, contributed reagents, and supervision. DN: conceptualization, interpreted data, contributed reagents, obtained funding, wrote paper, and supervised study. All authors read, edited, and finally approved manuscript.

## Conflict of Interest

The authors declare that the research was conducted in the absence of any commercial or financial relationships that could be construed as a potential conflict of interest.
