# Supplementary material for: Regulatory Roles of Drosophila Insulin-Like Peptide 1 (DILP1) in Metabolism Differ in Pupal and Adult Stages
Source: Front Endocrinol (Lausanne). 2020 Apr 21;11:180. doi: 10.3389/fendo.2020.00180 (PMC7186318; doi:10.3389/fendo.2020.00180)
Supplement: Supplementary file 1 [file Data_Sheet_1.PDF]

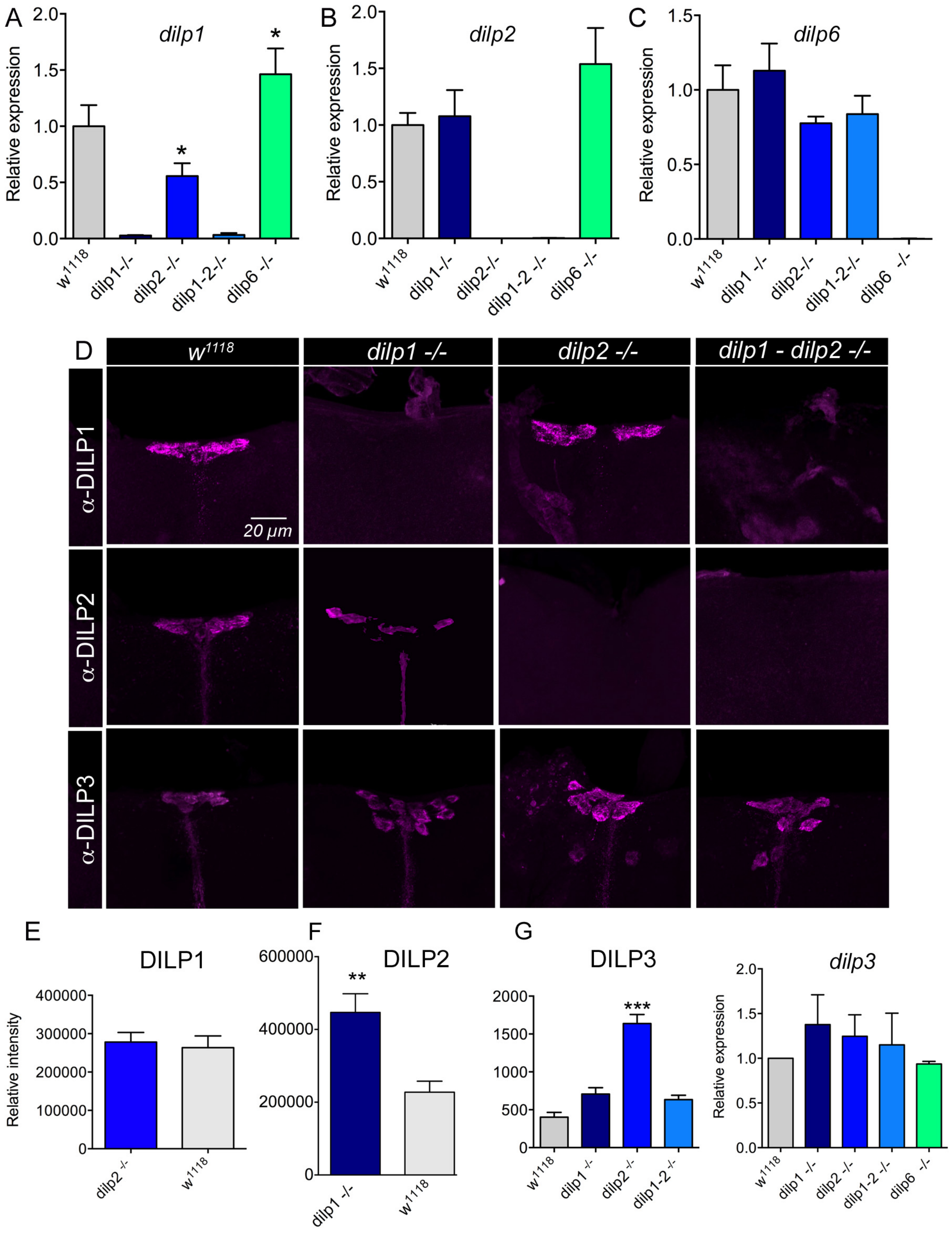

Supplementary Figure 1. Evaluation of mutant efficiency. **A**. qPCR reveals that in stage P8-9 pupae the *dilp1* and *dilp1/dilp2* mutants display *dilp1* levels that are close to zero, whereas in the *dilp6* mutant *dilp1* is

upregulated and in *dilp2* mutant slightly reduced. **B.** In the *dilp2* and *dilp1/dilp2* mutants *dilp2* levels are not detectable. **C.** The *dilp6* levels are only affected in the *dilp6* mutants. Data are presented as means ± S.E.M, n = 6 replicates for each genotype with 6 pupae in each replicate. (*$p < 0.05$, compared with w^{1118} flies, unpaired Students' t-test). **D.** Using immunocytochemistry with antisera to DILP1-3 it can be shown that labeling of IPCs in 1-week-old female flies is not detectable for anti-DILP1 in *dilp1* and double mutants and for DILP2 in *dilp2* and double mutants. DILP3 is upregulated in *dilp2* mutants. **E-G**. Quantification of immunofluorescence in female flies shows that DILP1 labeling is not affected in *dilp2* mutants (E), DILP2 is increased in *dilp1* mutants (F) and DILP3 strongly increased only in *dilp2* mutants (G). Data are presented as means ± S.E.M, n = 9-12 flies from 3 replicates. (**$p < 0.01$, ***$p < 0.001$, compared with w^{1118} flies, unpaired Students' t-test).

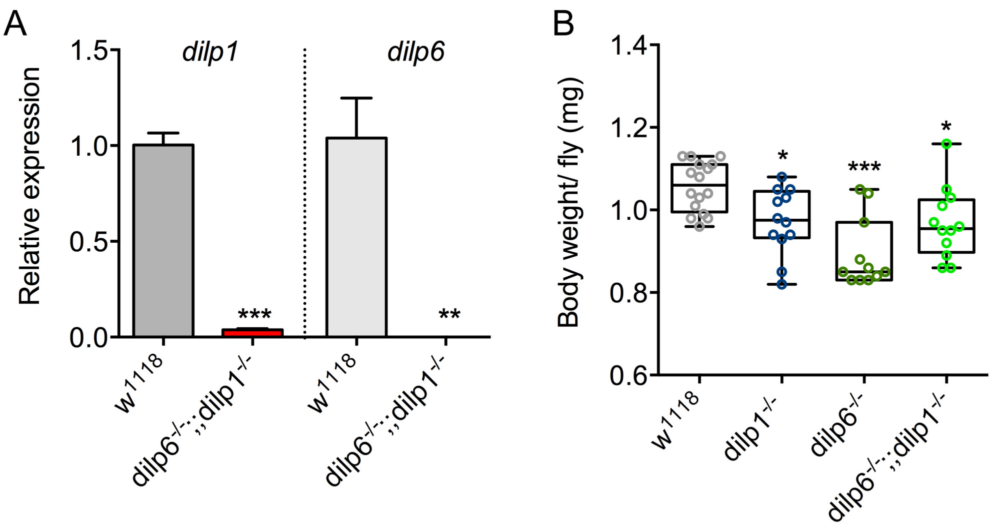

Supplementary Figure 2. Recombinant *dilp1/dilp6* mutant flies display reduced body mass. **A.** Transcripts of *dilp1* and *dilp6* in one-day-old *dilp1/dilp6* mutant flies. Data are presented as means ± S.E.M, n = 3 replicates for each genotype with 6 pupae in each replicate. (**$p < 0.001$, ***$p < 0.0001$, compared with w^{1118} flies, unpaired Students' t-test). **B.** Body weights are significantly reduced in single mutants and recombinant double mutants, but no additive effect of the double mutation was detected. (n = 11–16 flies per genotype from three replicates, One-way ANOVA followed by Tukey's test).

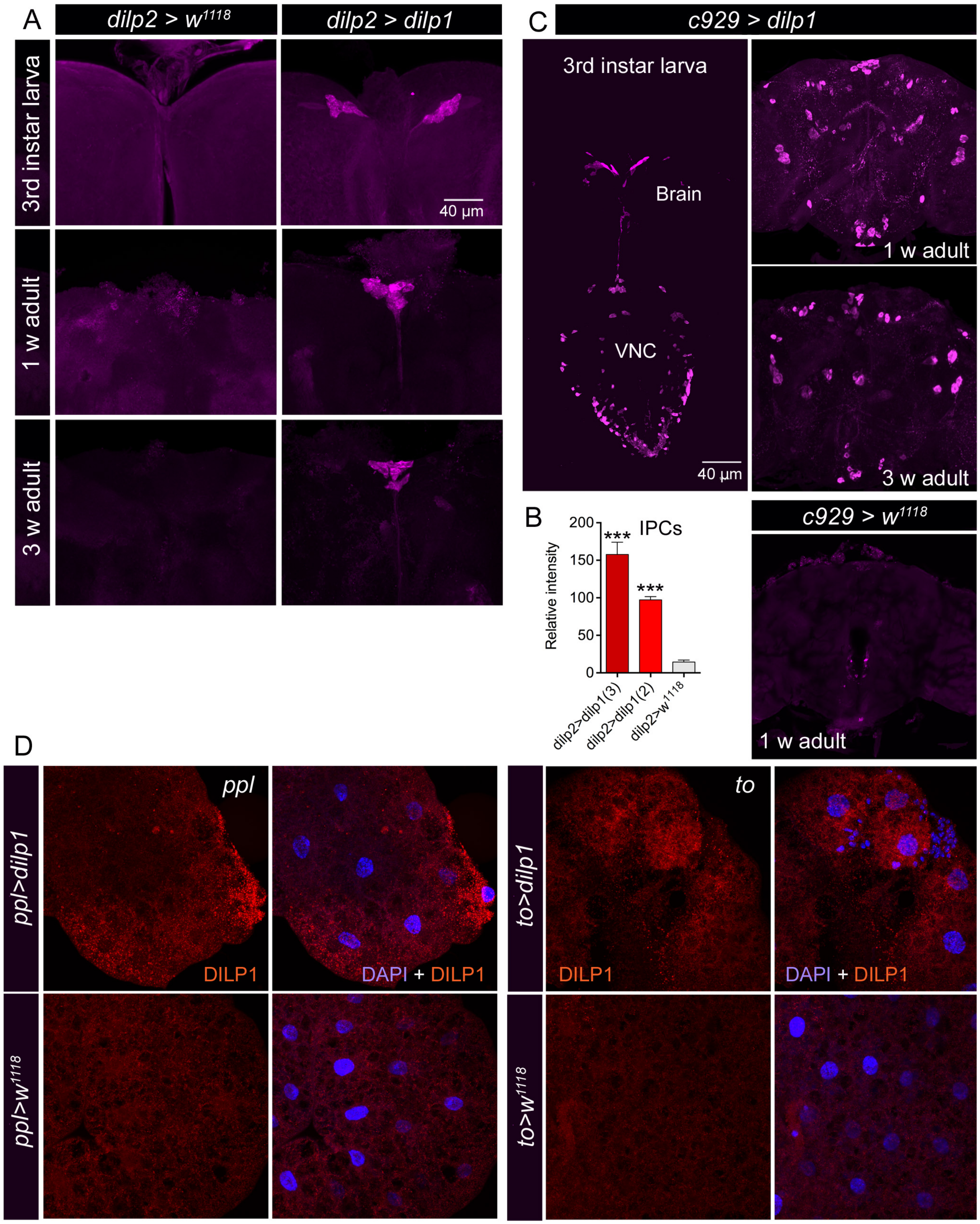

Supplementary Figure 3. Verification of ectopic *dilp1* expression by DILP1 immunolabeling. **A.** After *dilp2*-Gal4-driven *dilp1* expression strong DILP1 immunolabeling can be detected in IPCs of 3rd instar larvae as well as 1 and 3 week old adults, but not in controls (*dilp2*>w^{1118}). **B.** Quantification of DILP1 immunofluorescence in IPCs of one-week-old adults, using two different UAS-*dilp1* (2 and 3). Data are presented as means ±

S.E.M, n = 5-7 flies from 3 replicates. (***p < 0.001, compared with control flies, unpaired Students' t-test). **C.** Using the c929 driver DILP1 immunolabeling can be detected in numerous neuroendocrine cells in the CNS of larvae and brain of adults, but not in controls (c929>w^{1118}). **D.** Using two different fat body Gal4 drivers (*ppl* and *to*), DILP1 immunolabeling can be detected in adipocytes.

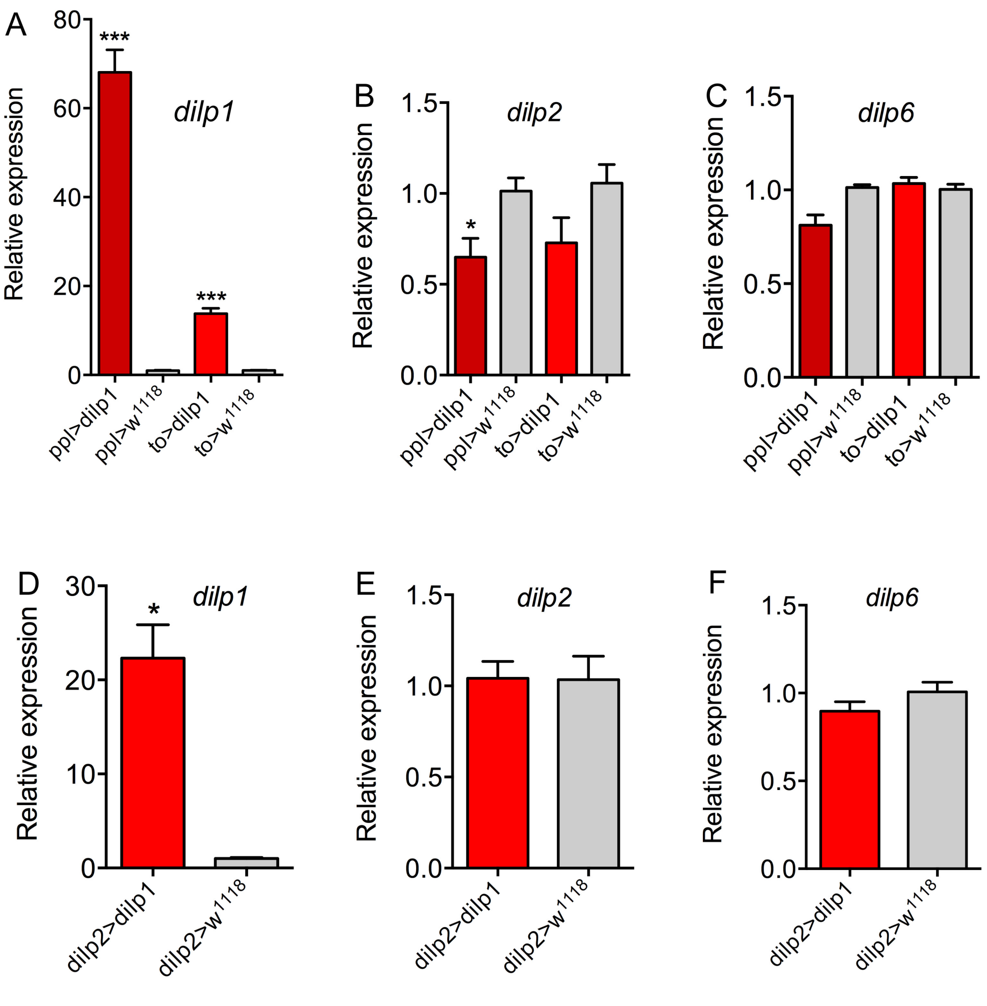

Supplementary Figure 4. Verification of ectopic *dilp1* expression by qPCR in stage P8-9 pupae. **A.** Using the fat body Gal4 drivers *ppl* and *to* a drastic increase of *dilp1* transcript was seen. **B.** The *dilp2* level was diminished after ppl-driven *dilp1*. **C.** No significant effect was seen on *dilp6* levels after *dilp1* expression. **D-F.** Driving *dilp1* in IPCs with *dilp2*-Gal4 drastically increases *dilp1*, but has no effect on *dilp2* or *dilp6*. Data are presented as means ± S.E.M, n = 5-6 replicates per genotype with 10 pupae in each replicate. (*p < 0.05, **p < 0.01, ***p < 0.01, compared with w^{1118} flies, unpaired Students' t-test).

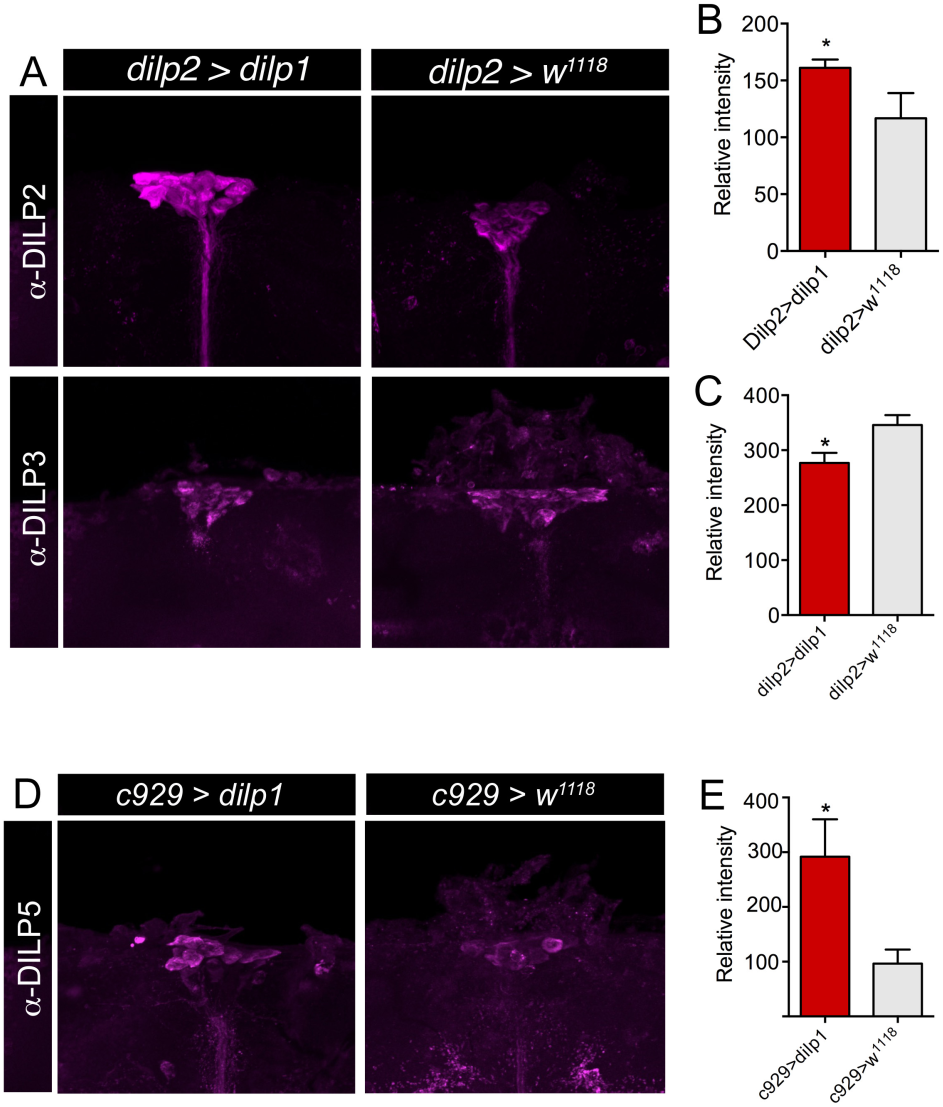

Supplementary Figure 5. Effects of ectopic *dilp1* expression on peptide levels of DILPs in one-week-old adults. **A**. Expressing *dilp1* in IPCs (*dilp2>dilp1*) increases DILP2 immunolabeling and decreases DILP3. **B** and **C**. Quantification of immunolabeling. Data are presented as means ± S.E.M, n = 7-10 per genotype from 3 replicates. (**p < 0.01, compared with w^{1118} flies, unpaired Students' t-test)**. D.** Using the broader *c929*-Gal4 to drive *dilp1* the DILP5 immunolabeling of IPCs increase. **E.** Quantification of DILP5 immunolabeling. Data are presented as means ± S.E.M, n = 9-12 from 3 replicates. (**p < 0.01, compared with w^{1118} flies, unpaired Students' t-test)**.**

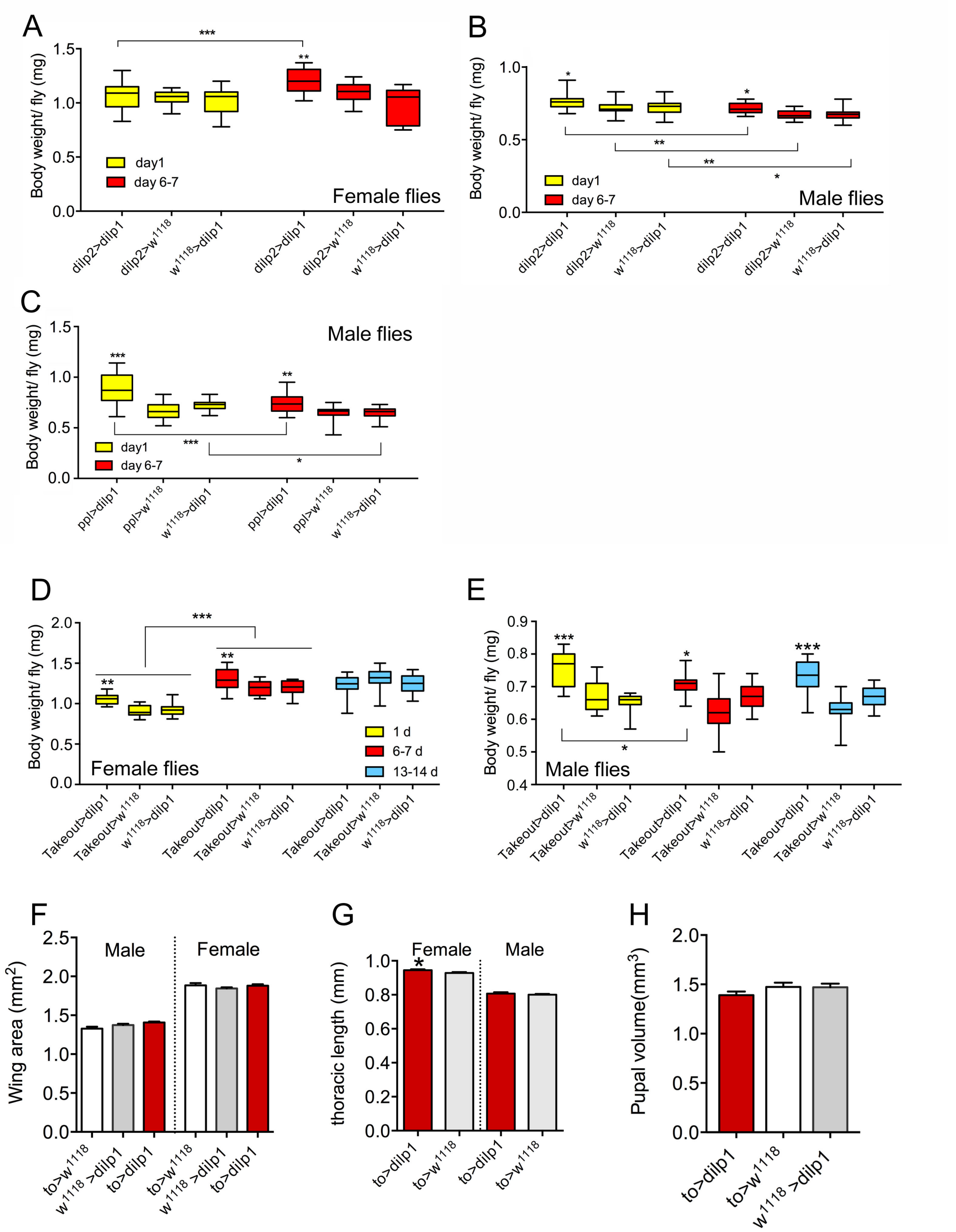

Supplementary Figure 6. Effects of ectopic *dilp1* expression on body weight and organismal size. **A.** Driving *dilp1* in IPCs with *dilp2*-Gal4 in females increases the weight compared to controls in older flies. Data are presented as medians ± range, n = 14–23 flies from three independent replicates (*p < 0.05, **p < 0.01, two-way ANOVA followed with Tukey's test). **B**. Driving *dilp1* in IPCs increases the weight of one-day-old and 6-7 day old male flies, compared to both controls. Furthermore the younger flies weigh more than the older ones for all genotypes. Data are presented as medians ± range, n = 14–24 flies per genotype from three independent replicates (**p < 0.01, ***p < 0.001, two-way ANOVA followed with Tukey's test). **C.** Expressing *dilp1* in the fat body (*ppl*-Gal4) of male flies leads to increased weight compared to controls in both young and older flies. However, in contrast to female flies, shown in Fig. 2K, there is no gain in weight over the first 5-6 days as adults, rather a decrease. Data are presented as medians ± range, n = 14–25 flies from three independent replicates (*p < 0.05, **p < 0.01, ***p < 0.001, two-way ANOVA followed with

Tukey's test). **D** and **E.** Using *to*-Gal4 the body masses show the same patterns as with *ppl*-Gal4 (Fig. 5C and S6C Fig), where body masses increase after *dilp1* over expression, and in females there is an additional weight gain over the first 5-6 days. The following days (13-14 d) no additional increase is seen. Data are presented as medians ± range, n = 9–27 flies per genotype from three independent replicates (*p < 0.05, **p < 0.01, ***p < 0.001, two-way ANOVA followed with Tukey's test). **F-H.** The *dilp1* expression obtained with the *to*-Gal4 does not result in a significant increase in wing area, (n = 16–22 flies per genotype from three replicates, One-way ANOVA followed with Tukey's test), whereas thorax length increased slightly (n = 19-32 flies per genotype (*p < 0.05 unpaired Students' t-test), but no effect on pupal volume (n = 29 flies per genotype from three replicates, One-way ANOVA followed with Tukey's test).

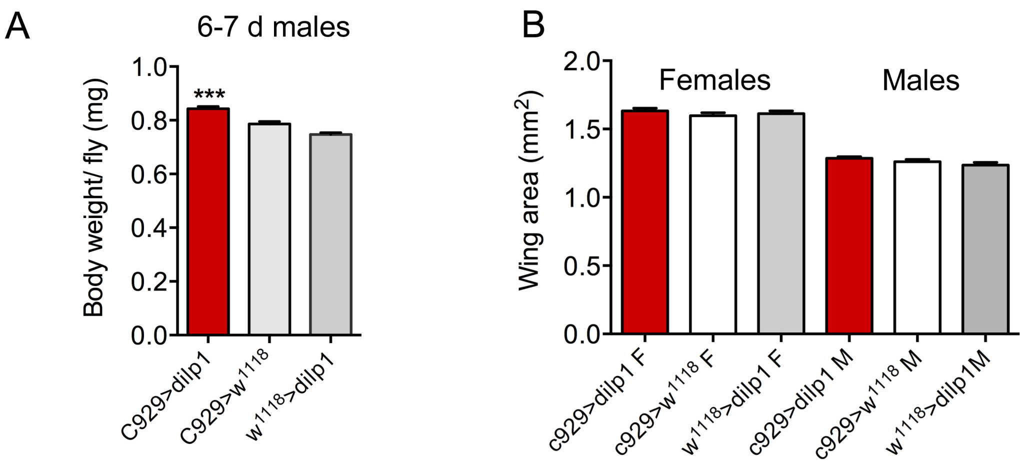

Supplementary Figure 7. Effects of *dilp1* expression on weight and wing area. **A** The body weight increased in male flies after ectopic *dilp1* expression with *c929*-Gal4, *** p<0.001, data are presented as means ± S.E.M, n = 16–29 flies per genotype from three independent replicates (One-way ANOVA followed with Tukey's test). **B**. The wing area is not affected by *c929*-driven *dilp1* expression. Data are presented as means ± S.E.M, n = 15 flies from three independent replicates (One-way ANOVA followed by Tukey's test). Data are presented as means ± S.E.M, n = 24 flies from three independent replicates (two-way ANOVA followed with Tukey's test).

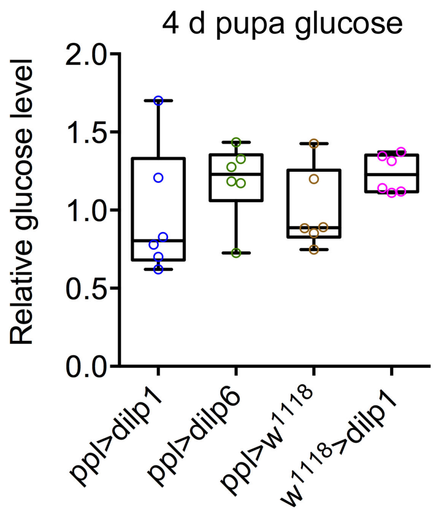

Supplementary Figure 8. Glucose levels in 4 d old pupae. Experiment performed as in Figure 3 D-F. We used 6 replicates per genotype with 4 pupae in each replicate (no significant changes; one-way ANOVA followed by Tukey's test).

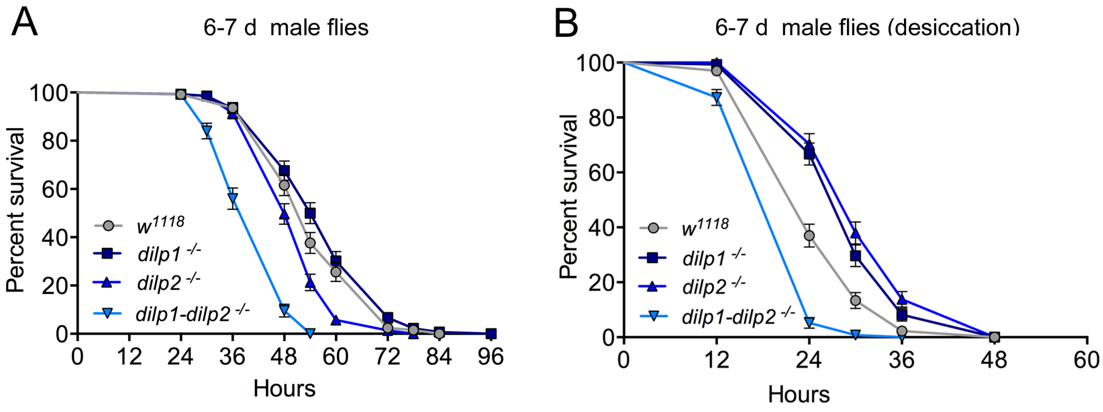

Supplementary Figure 9. Effect on starvation and desiccation in male *dilp* mutant flies. **A.** In 6-7 days old male flies *dilp1-dilp2* mutants are least resistant to starvation ($p<0.001$), followed by *dilp2* mutants ($p<0.001$), whereas *dilp1* mutants perform as controls; n = 125-141 flies from three independent replicates. However 6-7 d female flies perform as 3 d virgin females (see (Post et al., 2019) and Table 1). **B.** In males double mutants are less ($p<0.001$), and the other two mutants more resistant ($p<0.001$) to desiccation than controls, n = 134-135 flies from three independent replicates. Data are presented in survival curves and the error bars means S.E.M, as assessed by log-rank (Mantel–Cox) test].

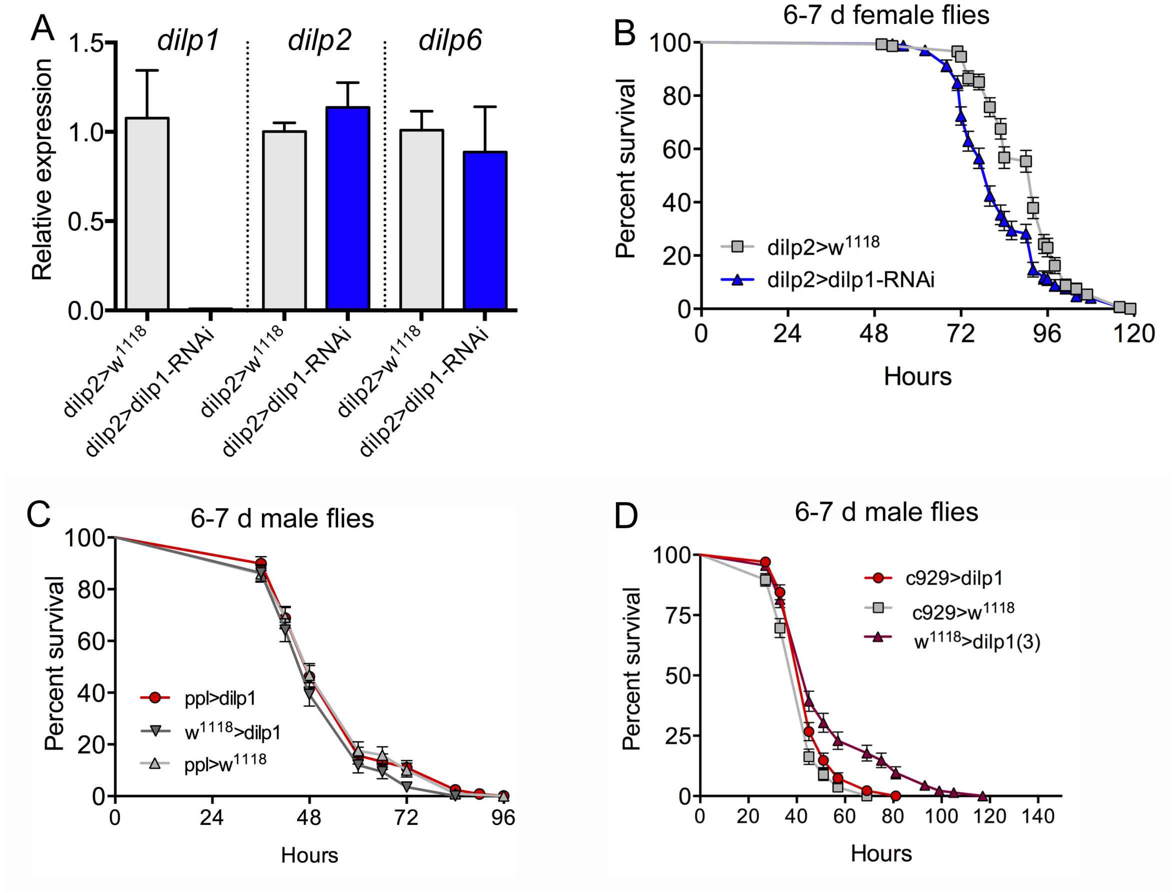

Supplementary Figure 10. Targeted *dilp1*-RNAi in IPCs reduces survival in flies exposed to starvation. **A.** The efficiency of *dilp2>dilp1*-RNAi on *dilp1* levels was monitored by qPCR. A strong reduction in *dilp1* was noted, but no effect was seen on levels of *dilp2* or *dilp6*. Data are presented as means ± S.E.M, n = 3 replicates per genotype with 10 pupae in each replicate. (*p < 0.05, compared with control flies, unpaired Students' t-test). **B.** In newly eclosed female flies *dilp2>dilp1*-RNAi resulted in reduced survival during starvation. n = 148-170 flies from three independent replicates. Data are presented in survival curves and the error bars means S.E.M [***p < 0.001, as assessed by log-rank (Mantel–Cox) test]. **C.** In 6-7 d old males *dilp1* overexpression in fat body (*ppl*-Gal4) has no effect on starvation response. n = 117-128 flies from three independent replicates. **E**. c929-driven *dilp1* does not affect the response to starvation, n = 132-135 flies per genotype from three independent replicates.
